# COVID-19 vaccination, risk-compensatory behaviours, and contacts in the UK

**DOI:** 10.1038/s41598-023-34244-2

**Published:** 2023-05-25

**Authors:** John Buckell, Joel Jones, Philippa C. Matthews, Sir Ian Diamond, Emma Rourke, Ruth Studley, Duncan Cook, Ann Sarah Walker, Koen B. Pouwels, the COVID-19 Infection Survey Team, Tina Thomas, Daniel Ayoubkhani, Russell Black, Antonio Felton, Megan Crees, Joel Jones, Lina Lloyd, Esther Sutherland, Emma Pritchard, Karina-Doris Vihta, George Doherty, James Kavanagh, Kevin K. Chau, Stephanie B. Hatch, Daniel Ebner, Lucas Martins Ferreira, Thomas Christott, Wanwisa Dejnirattisai, Juthathip Mongkolsapaya, Sarah Cameron, Phoebe Tamblin-Hopper, Magda Wolna, Rachael Brown, Richard Cornall, Gavin Screaton, Stuart Cox, Kevin Paddon, Tim James, Thomas House, Julie Robotham, Paul Birrell, Helena Jordan, Tim Sheppard, Graham Athey, Dan Moody, Leigh Curry, Pamela Brereton, Ian Jarvis, Anna Godsmark, George Morris, Bobby Mallick, Phil Eeles, Jodie Hay, Harper VanSteenhouse, Jessica Lee, Sean White, Tim Evans, Lisa Bloemberg, Katie Allison, Anouska Pandya, Sophie Davis, David I. Conway, Margaret MacLeod, Chris Cunningham, Katrina Lythgoe, David Bonsall, Tanya Golubchik, Helen Fryer

**Affiliations:** 1 Health Economics Research Centre, Richard Doll Building, Old Road Campus, Nuffield Department of Population Health, University of Oxford, Oxford, OX3 7LF UK; 2 Health Behaviours, Nuffield Department of Primary Health Care Sciences, University of Oxford, Oxford, UK; 3 The National Institute for Health Research Oxford Biomedical Research Centre, University of Oxford, Oxford, UK; 4 Office for National Statistics, Newport, UK; 5 The Francis Crick Institute, 1 Midland Road, London, NW1 1AT UK; 6 Division of Infection and Immunity, University College London, Gower St, London, WC1E 6BT UK; 7 Department of Infection, University College London Hospitals, 235 Euston Rd, London, NW1 2BU UK; 8 Nuffield Department of Medicine, University of Oxford, Oxford, UK; 9 The National Institute for Health Research Health Protection Research Unit in Healthcare Associated Infections and Antimicrobial Resistance at the University of Oxford, Oxford, UK; 10 MRC Clinical Trials Unit at UCL, UCL, London, UK; 11 Oxford University Hospitals NHS Foundation Trust, Oxford, UK; 12 University of Manchester, Manchester, UK; 13 Health Improvement Directorate, Public Health England, London, UK; 14 IQVIA, London, UK; 15 National Biocentre, Milton Keynes, UK; 16 Glasgow Lighthouse Laboratory, London, UK; 17 Department of Health and Social Care, London, UK; 18 Welsh Government, Cardiff, UK; 19 Scottish Government, Edinburgh, UK; 20 Public Health Scotland, Edinburgh, UK; 21 Big Data Institute, Nuffield Department of Population Health, University of Oxford, Oxford, UK

**Keywords:** Viral infection, Epidemiology, Human behaviour

## Abstract

The physiological effects of vaccination against SARS-CoV-2 (COVID-19) are well documented, yet the behavioural effects not well known. Risk compensation suggests that gains in personal safety, as a result of vaccination, are offset by increases in risky behaviour, such as socialising, commuting and working outside the home. This is potentially important because transmission of SARS-CoV-2 is driven by contacts, which could be amplified by vaccine-related risk compensation. Here, we show that behaviours were overall unrelated to personal vaccination, but—adjusting for variation in mitigation policies—were responsive to the level of vaccination in the wider population: individuals in the UK were risk compensating when rates of vaccination were rising. This effect was observed across four nations of the UK, each of which varied policies autonomously.

## Introduction

The United Kingdom was the first country to begin a national COVID-19 vaccination programme on 8 December 2020, and as of 16th January 2023, 94% of the population (aged 12 and over) had received a first vaccination^1^.

In the UK, the most commonly used vaccines are the BNT162b2 messenger RNA (mRNA) vaccine (Pfizer-BioNTech), the mRNA-1273 (Moderna) vaccine, and the Oxford-AstraZeneca adenovirus vector vaccine, ChAdOx1 nCOV-19 (denoted here ChAdOx1). These vaccines are effective in preventing (symptomatic) SARS-CoV-2 infection and hospitalisation and deaths^2–14^.

Evidence is limited on whether SARS-CoV-2 vaccination induces a behavioural, as well as physiological, response in individuals. Individuals who have been immunised may be less fearful about contracting SARS-CoV-2, getting severely ill from infection, or spreading the virus when infected. In turn, they may begin to interact with others more often and/or less cautiously. This type of behaviour is known as risk compensation or the Peltzman effect^15^, and has been studied in various health settings with mixed evidence on its presence^16–18^. In the context of SARS-CoV-2, there is scepticism regarding risk compensation for face coverings^19^, but concern that risk compensation may pervade post-vaccination behaviour^20,21^. That is, while the link between vaccination and risk compensation has been discussed in prior literature, comprehensive empirical evidence on the potential (absence of) changes in behaviour upon vaccination of oneself, vulnerable household members, or population-level vaccination uptake (initially reflective of vaccination of vulnerable populations and healthcare workers) is lacking.

Risk compensation could present significant short- and medium-term public health risks, particularly if individuals—or their unvaccinated household members—change their behaviour before being fully protected by vaccination or when protection from vaccination is incomplete or temporary. Misconceptions about the extent of protection after COVID-19 vaccination might be common, as exemplified by the Dutch health minister advocating that one could go partying one day after getting a single dose of the Ad26.COV2.S (Janssen) vaccine^22^. Developing an understanding of the extent to which post-vaccination behaviours are risk-compensatory is important for public health messaging and policy development. Two studies did not find that behaviours of individuals—compliance with COVID guidelines, mask wearing, and social distancing—were related to vaccination^23,24^. A further study did find vaccinated individuals reported more social contacts than unvaccinated individuals^25^. However, individuals that decide to vaccinate are different in many ways compared to those that decide to not get vaccinated, and hence including individuals that remain unvaccinated throughout is unlikely to lead to estimation of potential risk-compensatory behaviour. To overcome this issue, we restricted our analyses evaluating potential changes in behaviour upon getting vaccinated to individuals that ever get vaccinated, which is more likely to lead to causal estimates. In addition, we are able to build in existing evidence with larger data and a wider set of outcomes. Thus, our first research question is whether individual behaviours changed following their own vaccination.

These studies did not explore potential risk compensation following vaccination of frail household members. However, it could be hypothesised that some individuals that consider themselves at low risk of getting adverse consequences of infection may be more likely to change their behaviour when frail individuals in their network are protected through vaccination than to change their behaviour when they receive their own vaccination. Our second research question is whether individual behaviours of those that live with frail individuals changed following vaccination of frail household members.

Prior studies did measure response to population-level uptake but were either based on non-representative samples of the population or did not follow individuals over time^23,24^. Our third research question is whether individual behaviours responded to population-level vaccination.

Here, we use a much larger, representative sample from the UK with longitudinal follow-up of individuals to evaluate the potential impact of individuals’ own vaccination, uptake of vaccination within the household, and regional level of vaccination uptake on a rich set of behavioural outcomes. Three samples are used for three analyses: individuals who were at any time vaccinated, those who were unvaccinated but in households with vulnerable individuals, and the entire population. We used the Office for National Statistics (ONS) COVID-19 Infection Survey (CIS)—a large, community-based survey of individuals aged 2 years and older living in randomly selected households across the United Kingdom. We investigate risk-compensatory behaviours in 10 reported outcomes, including physical and socially-distanced contacts (each divided into three categories according to the age of the contact), time that others spend in individuals’ homes, individuals’ time in other people’s houses, location of work (home, outside the home, combination), and private vs. public transport for commuting.

## Results

### Behaviour changes among vaccinated individuals aged 18–64 years

This analysis included observations between 1st October 2020 and 15th September 2021 representing 1,839,911 visits of 161,309 individuals aged 18–64 years from 102,260 households who did not report working in a patient-facing healthcare role. 18 years of age was the minimum age at the time of analysis. Individuals were only included if there was evidence that they were vaccinated at any point during the survey to avoid that those not willing to get vaccinated, who are expected to have different behaviours than those that are willing to get vaccinated. We excluded over-65s (because patterns of response to vaccination were different insofar as individuals had fewer contacts) and those reporting working in patient-facing healthcare (see Supplementary Figs. 1–3). Characteristics of included individuals are summarized in Table 1.

**Table 1:** Descriptive statistics of the characteristics of individuals included in analyses at the first visit on or after 1 October 2020.

	Own vaccination 18–64y N = 161,309				Household vaccination 18–64y N = 7626				Population vaccination 2 + years N = 501,679			
	Mean	s.d	Min	Max	Mean	s.d	Min	Max	Mean	s.d	Min	Max
Age	45.57	12.44	18.00	64.00	46.28	14.21	18.00	64.00	47.12	21.81	2.00	95.00
Female	0.53	0.50	0.00	1.00	0.53	0.50	0.00	1.00	0.53	0.50	0.00	1.00
Black	0.01	0.09	0.00	1.00	0.01	0.08	0.00	1.00	0.01	0.10	0.00	1.00
Asian	0.05	0.21	0.00	1.00	0.05	0.21	0.00	1.00	0.04	0.20	0.00	1.00
Mixed	0.01	0.12	0.00	1.00	0.02	0.13	0.00	1.00	0.02	0.13	0.00	1.00
Other	0.01	0.10	0.00	1.00	0.01	0.09	0.00	1.00	0.01	0.10	0.00	1.00
Country IMD Rank	58.14	27.06	0.00	100.00	58.98	26.23	0.02	100.00	58.33	27.00	0.00	100.00
North East	0.04	0.20	0.00	1.00	0.04	0.20	0.00	1.00	0.04	0.19	0.00	1.00
North West	0.12	0.33	0.00	1.00	0.12	0.33	0.00	1.00	0.12	0.32	0.00	1.00
Yorkshire & The Humber	0.09	0.28	0.00	1.00	0.08	0.28	0.00	1.00	0.08	0.28	0.00	1.00
East Midlands	0.07	0.25	0.00	1.00	0.07	0.25	0.00	1.00	0.07	0.25	0.00	1.00
West Midlands	0.08	0.27	0.00	1.00	0.09	0.28	0.00	1.00	0.08	0.27	0.00	1.00
East	0.11	0.31	0.00	1.00	0.11	0.32	0.00	1.00	0.10	0.30	0.00	1.00
London	0.20	0.40	0.00	1.00	0.17	0.38	0.00	1.00	0.18	0.39	0.00	1.00
South East	0.13	0.34	0.00	1.00	0.14	0.34	0.00	1.00	0.13	0.34	0.00	1.00
South West	0.08	0.26	0.00	1.00	0.09	0.28	0.00	1.00	0.08	0.28	0.00	1.00
N Ireland	0.02	0.14	0.00	1.00	0.02	0.14	0.00	1.00	0.02	0.12	0.00	1.00
Scotland	0.04	0.19	0.00	1.00	0.04	0.20	0.00	1.00	0.08	0.28	0.00	1.00
Wales	0.03	0.17	0.00	1.00	0.03	0.17	0.00	1.00	0.02	0.15	0.00	1.00
Household size 2	0.40	0.49	0.00	1.00	0.55	0.50	0.00	1.00	0.41	0.49	0.00	1.00
Household size 3	0.20	0.40	0.00	1.00	0.26	0.44	0.00	1.00	0.17	0.37	0.00	1.00
Household size 4	0.18	0.39	0.00	1.00	0.14	0.34	0.00	1.00	0.17	0.38	0.00	1.00
Household size 5	0.07	0.25	0.00	1.00	0.06	0.23	0.00	1.00	0.08	0.28	0.00	1.00
Multi-generation	0.06	0.24	0.00	1.00	0.05	0.22	0.00	1.00	0.07	0.25	0.00	1.00
Urban/rural 2	0.42	0.49	0.00	1.00	0.42	0.49	0.00	1.00	0.42	0.49	0.00	1.00
Urban/rural 3	0.10	0.30	0.00	1.00	0.11	0.31	0.00	1.00	0.10	0.31	0.00	1.00
Urban/rural 4	0.10	0.29	0.00	1.00	0.12	0.33	0.00	1.00	0.11	0.31	0.00	1.00
Long-term health condition	0.23	0.42	0.00	1.00	0.00	0.00	0.00	0.00	0.25	0.43	0.00	1.00

*IMD* index of multiple deprivation.

To ensure that time since vaccination would not be perfectly confounded with calendar time we assessed age-specific variation in uptake of the first vaccination. Supplementary Fig. 4 shows that there was substantial variation in the timing of first doses within the different age categories. The mean outcome values are presented in Table 2 and aggregated into binary variables of “zero [contacts]” or “any [contacts]” (the regression models used all outcome categories). On average, individuals were most likely to report having at least one socially-distanced contact with someone aged 18–69 years outside their household in the past 7 days (75.5% of visits). Among the risk behaviours considered, on average, individuals were least likely to report having used public transport for a commute (for work or study purposes) in the past 7 days (9.5% of visits). Individuals were slightly more likely to report that others visited their home in the past 7 days (40.8%) than they themselves visiting others’ homes in the past 7 days (36.4%). Individuals were more likely to report working at home than working outside of home over the past 7 days (46.6% reporting only working at home).

**Table 2:** Summaries of self-reported behaviours across all outcomes for in different analyses.

Outcome	Behaviour captured by binary variable	% of Any		
		Own vaccination 18–64y (%)	Household vaccination 18–64y (%)	Population vaccination 2 + years (%)
Physical contacts with under 18s	Any past 7-day contacts outside the household	23.5	22.4	29.7
Physical contacts with 18–69 years	Any past 7-day contacts outside the household	38.1	32.9	39.7
Physical contacts with over 70s	Any past 7-day contacts outside the household	17.6	14.9	18.3
Socially-distanced contacts with under 18s	Any past 7-day contacts outside the household	34.2	30.3	36.2
Socially-distanced contacts with 18–69 years	Any past 7-day contacts outside the household	75.5	72.1	72.2
Socially-distanced contacts with over 70s	Any past 7-day contacts outside the household	34.2	32.6	34.4
Visits to others' homes	Any past 7-day home visits	36.4	35.7	34.9
Others' visits to own home	Any past 7-day home visits	40.8	40.7	40.5
Working/studying outside home	Any past 7-day working outside home	53.4	53.8	56.8
Taking public transport to work/place of education	Any past 7-day public transport	9.5	8.7	11.0

The numerator is the number of observations for which “any” was reported and the denominator is the total number of observations in that analysis. For own vaccination, 23.5% of observations were reported as any past 7-day, outside of the household, physical contacts with under 18 year-olds. All contact variables relate to individuals outside the household. Denominators for work variables restricted to those reporting working or in education. Tables showing how these percentages were derived are provided in the supplementary materials.

**Figure 1:**
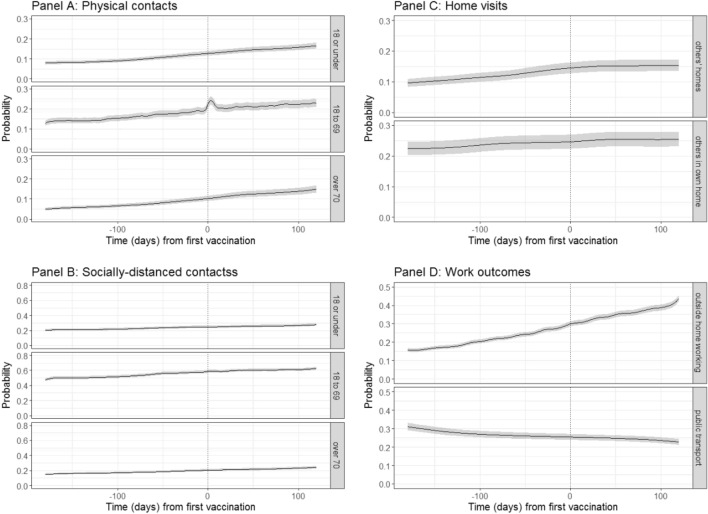
Probabilities of behavioural outcomes for individuals aged 18–64y by time from first vaccination, first dose. Top left (**A**): past 7-day reported physical, outside of household contacts; bottom left (**B**) past 7-day reported socially-distanced, outside of household contacts; top right (**C**) past 7-day reported home visits; bottom right (Panel D): past 7-day reported work outcomes for those that are working or in education. Dotted line shows day of own first vaccination. “18 or under”, “18 to 69” and “over 70” denote the ages of the people with whom individuals in the sample had contact.

We calculated the variation in 10 behavioural outcomes as a function of the time to individuals’ own first vaccination (Fig. 1), adjusting for multiple confounders, including household composition, individual characteristics, sociodemographic variables, and background prevalence using ordinal generalised additive models. Overall, reported socialising increased over time, with no observed change associated with vaccination.

The probability of any physical contacts increased with time since first vaccination for outside of the household contacts with under-18s, 18–69 year-olds, and over-70s (Fig. 1A). For physical contacts with either under-18s or over-70s, no difference in the pre- and post-vaccination gradients were observed; that is, there was no evidence of a behavioural response to being vaccinated. To illustrate, for physical contacts with under 18 year-olds, after adjusting for confounders including calendar time, the pre-vaccination gradient for 50 to 14 days prior to vaccination was 0.00048 [95% CI 0.00040–0.0057] meaning that for each 100 days, the probability of any physical contacts with under 18 year-olds would increase by 0.048. The post-vaccination gradient for the period 1–13 days after vaccination was 0.00047 [95% CI 0.00032–0.00058; difference of − 0.00004, 95% CI − 0.00019–0.00012 compared to 50–14 days before]. Thus, the gradients in the two periods appear to be almost identical. For physical contacts with 18–69 year-olds, the probability temporarily increased in the immediate period after the first vaccination (Fig. 1A; Table 3). However, this increase was only transient, with no evidence of difference in gradient before versus 14–50 days after vaccination (Fig. 1A; Table 3).

**Table 3:** Gradients and differences in gradients of outcomes over time between pre- and post-vaccination periods, individuals’ own vaccination (18–64y).

Outcome	Period compared	Gradient (probability)		Difference in gradients: percentiles of simulated distribution		
		Pre vaccination	Post vaccination	2.5	50	97.5
Physical contacts with under 18s	− 50 to − 14 days versus 1–13 days	0.00048	0.00047	− 0.00019	− 0.00004	0.00012
Physical contacts with 18–69 years	− 50 to − 14 days versus 1–6 days	0.00042	− 0.00025	− 0.00195	− 0.00067	0.00059
Physical contacts with 18–69 years	− 50 to − 14 days versus 7–13 days	0.00042	− 0.00367	− 0.00475	− 0.00367	− 0.00264
Physical contacts with over 70s	− 50 to − 14 days versus 1–13 days	0.00041	0.00053	− 0.00003	0.00012	0.00026
Socially-distanced contacts with under 18s	− 50 to − 14 days versus 1–13 days	0.00029	0.00014	− 0.00037	− 0.00015	0.00007
Socially-distanced contacts with 18–69 years	− 50 to − 14 days versus 1–13 days	0.00038	− 0.00020	− 0.00100	− 0.00057	− 0.00014
Socially-distanced contacts with over 70s	− 50 to − 14 days versus 1–13 days	0.00042	0.00029	− 0.00030	− 0.00013	0.00005
Visits to others' homes	− 50 to − 14 days versus 1–13 days	0.00028	0.00016	− 0.00021	− 0.00012	0.00000
Others' visits to own home	− 50 to − 14 days versus 1–13 days	0.00003	0.00016	− 0.00001	0.00013	0.00027
Working/studying outside home	− 50 to − 14 days versus 1–13 days	0.00093	0.00063	− 0.00018	0.00031	0.00079
Taking public transport to work/place of education	− 50 to − 14 days versus 1–13 days	− 0.00012	− 0.00012	− 0.00022	− 0.00001	0.00023
Physical contacts with under 18s	− 50 to − 14 days versus 14–50 days	0.00048	0.00047	− 0.00012	− 0.00001	0.00009
Physical contacts with 18–69 years	− 50 to − 14 days versus 14–50 days	0.00042	0.00018	− 0.00048	− 0.00024	0.00000
Physical contacts with over 70s	− 50 to − 14 days versus 14–50 days	0.00041	0.00042	− 0.00009	0.00000	0.00001
Socially-distanced contacts with under 18s	− 50 to − 14 days versus 14–50 days	0.00029	0.00024	− 0.00018	− 0.00004	0.00009
Socially-distanced contacts with 18–69 years	− 50 to − 14 days versus 14–50 days	0.00038	0.00045	− 0.00013	0.00007	0.00027
Socially-distanced contacts with over 70s	− 50 to − 14 days versus 14–50 days	0.00042	0.00022	− 0.00031	− 0.00019	− 0.00008
Visits to others' homes	− 50 to − 14 days versus 14–50 days	0.00028	0.00007	− 0.00028	− 0.00021	− 0.00013
Others' visits to own home	− 50 to − 14 days versus 14–50 days	0.00003	0.00013	− 0.00001	0.00010	0.00021
Working/studying outside home	− 50 to − 14 days versus 14–50 days	0.00093	0.00113	− 0.00042	− 0.00019	0.00003
Taking public transport to work/place of education	− 50 to − 14 days versus 14–50 days	–0.00012	− 0.00014	− 0.00022	− 0.00002	0.000188

Percentiles of the distribution of 10,000 simulations from the Bayesian posterior distributions of the fitted GAM models for each outcome. Pre-vaccination was 50–14 days prior to first vaccination. Post-vaccination periods were 1–13 days (upper table) and 14–50 days (lower table). For 18–69 year-old physical contacts, the pre-period was divided into two parts to reflect the peak in probability around the time of vaccination. An example for interpretation: for physical contacts with under 18 year-olds, the pre-vaccination gradient for 50–14 days prior to vaccination was 0.0004 meaning that for each 100 days, the probability of any physical contacts with under 18 year-olds would increase by 0.04.

There were no differences between the gradients of the pre- and post-vaccination periods in terms of reporting any socially-distanced contacts with others outside the household (Fig. 1B; Table 3), the probability of reporting any visits to others’ home or others’ visits to own home (Fig. 1C; Table 3), or the probability of reporting working from home and using public transport for commuting (Fig. 1D; Table 3). This was, broadly, the case across the ten behaviours shown in Fig. 1 for the comparison of post-vaccination periods with pre-vaccination. Thus, there was no evidence of a behavioural response to being vaccinated on these outcomes.

In complementary analyses, we fitted interval-censored regressions following the same procedure as above for variables that were recorded in categories. By using interval-censored regression we could estimate the number of contacts over time, instead of the probability of falling into a specific category in the ordinal regressions. A general trend of increasing contacts and household visits was observed across outcomes. However, no evidence of a behavioural response to vaccination was observed. Results are presented in Supplementary Figs. 5.

### Influence of vaccination of vulnerable household members on behaviour of unvaccinated 18–64 year olds

**Figure 2:**
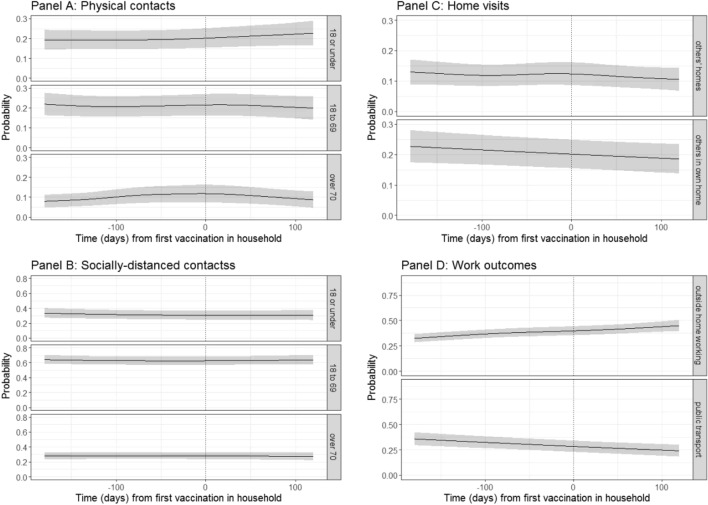
Probabilities of behavioural outcomes for unvaccinated individuals aged 18–64y by time to vaccination of the first vulnerable person in the household, first dose. Top left (**A**): past 7-day reported physical, outside of household contacts; bottom left (**B**): past 7-day reported socially-distanced, outside of household contacts; top right (**C**): past 7-day reported home visits; bottom right (**D**): past 7-day reported work outcomes for those that are working or in education. Dotted line shows day of own first vaccination. “18 or under”, “18 to 69” and “over 70” denote the ages of the people with whom individuals in the sample had contact.

This analysis included observations between 1st October 2020 and 15th September 2021 representing 53,846 visits of 7626 unvaccinated individuals aged 18–64 years old without long-term health conditions and who did not report working in patient-facing healthcare roles from 6958 households who had at least one vulnerable household member (defined as aged 65 and over or had a long-term health condition, in keeping with vaccine allocation groups of the UK government^26^). Characteristics of included individuals are summarized in Table 1. Unvaccinated individuals were included only up until they had themselves been vaccinated, i.e. examining how individuals living with vulnerable household members reacted to the vulnerable household members being vaccinated, before they themselves were vaccinated. We calculated the variation in 10 behavioural outcomes of these individuals, adjusting for multiple confounders including household composition, individual characteristics, sociodemographic variables; and background prevalence (Fig. 2). There is some evidence to suggest that physical contacts with under-18s increased following the first vaccination of the last vulnerable household member (Fig. 2A). Otherwise, no meaningful difference in the trend was observed between the probability of physical contacts and time to the last vulnerable person in the household having their first vaccination. We found no evidence that trends in other reported behaviours changed upon vaccination of the last vulnerable household member in the household (Fig. 2B–D).

In interval-censored regressions, the number of contacts and household visits appeared to be stable over time to the last vulnerable person in the household having their first vaccination. No evidence of a behavioural response to vaccination was observed. Results are presented in Supplementary Fig. 6.

### Influence of population-level vaccine uptake

This analysis included observations between 1st October 2020 and 15th September 2021 representing 4,508,755 visits of 501,679 individuals aged 2–95 years old from 238,641 households. Data on vaccination rates were only available for England and Scotland for the entire period; individuals from Northern Ireland and Wales are excluded from analyses. Characteristics of included individuals are summarized in Table 1.

**Figure 3:**
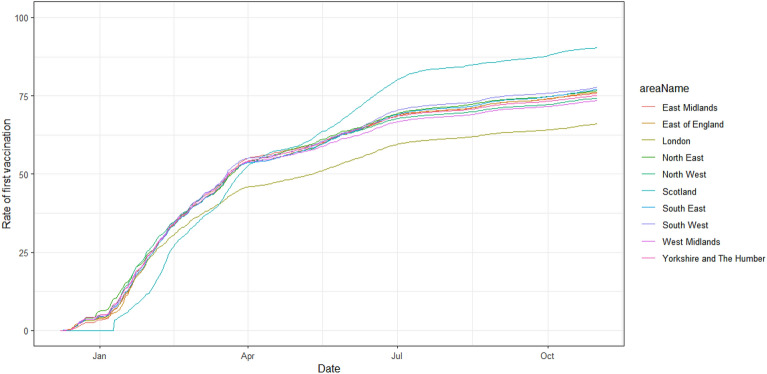
Percentage population SARS-CoV-2 vaccine uptake (first dose) over time during 2021 in England and Wales (split by region in England). Source: UK government official statistics, 2021a.

Population rates of first vaccination (taken from administrative sources) increased over time during 2021 across regions in England and Scotland (Fig. 3). Vaccination rates increased earlier in the English regions compared to the other countries in the UK, but from April until October 2021, the percentages were highest in Wales and Scotland, with vaccination rates in London appearing to lag behind other parts of the UK. While there are considerable uncertainties about the actual number of individuals eligible in each area, this is the information that is provided by the Government and the source individuals may act upon if they are responsive to population-level vaccine uptake information.

**Figure 4:**
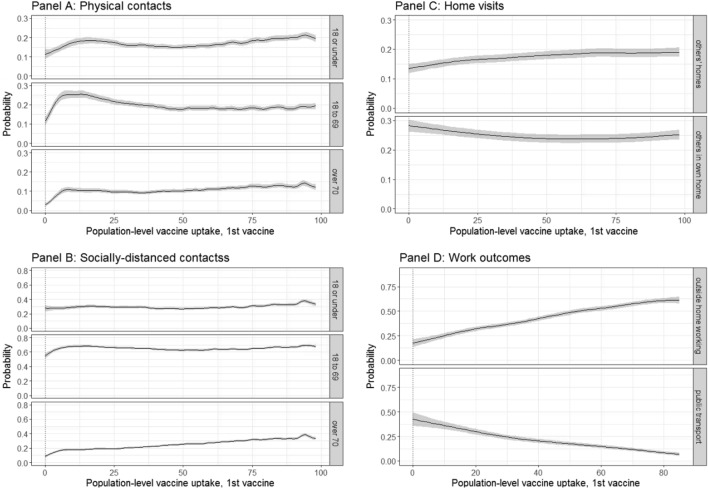
Probabilities of behavioural outcomes by population level vaccination %, first dose. Top left (**A**): past 7-day reported physical, outside of household contacts; bottom left (**B**): past 7-day reported socially-distanced, outside of household contacts; top right (**C**): past 7-day reported home visits; bottom right (**D**): past 7-day reported work outcomes for those that are working or in education. “18 or under”, “18 to 69” and “over 70” denote the ages of the people with whom that individuals in the sample had contact.

We calculated the variation in 10 behavioural outcomes as a function of the percentage of people who had had a first vaccination in the local population (defined as CIS geographical regions), using all individuals in the survey (Fig. 4), adjusting for multiple confounders including household composition, individual characteristics, sociodemographic variables, and background prevalence.

**Figure 5:**
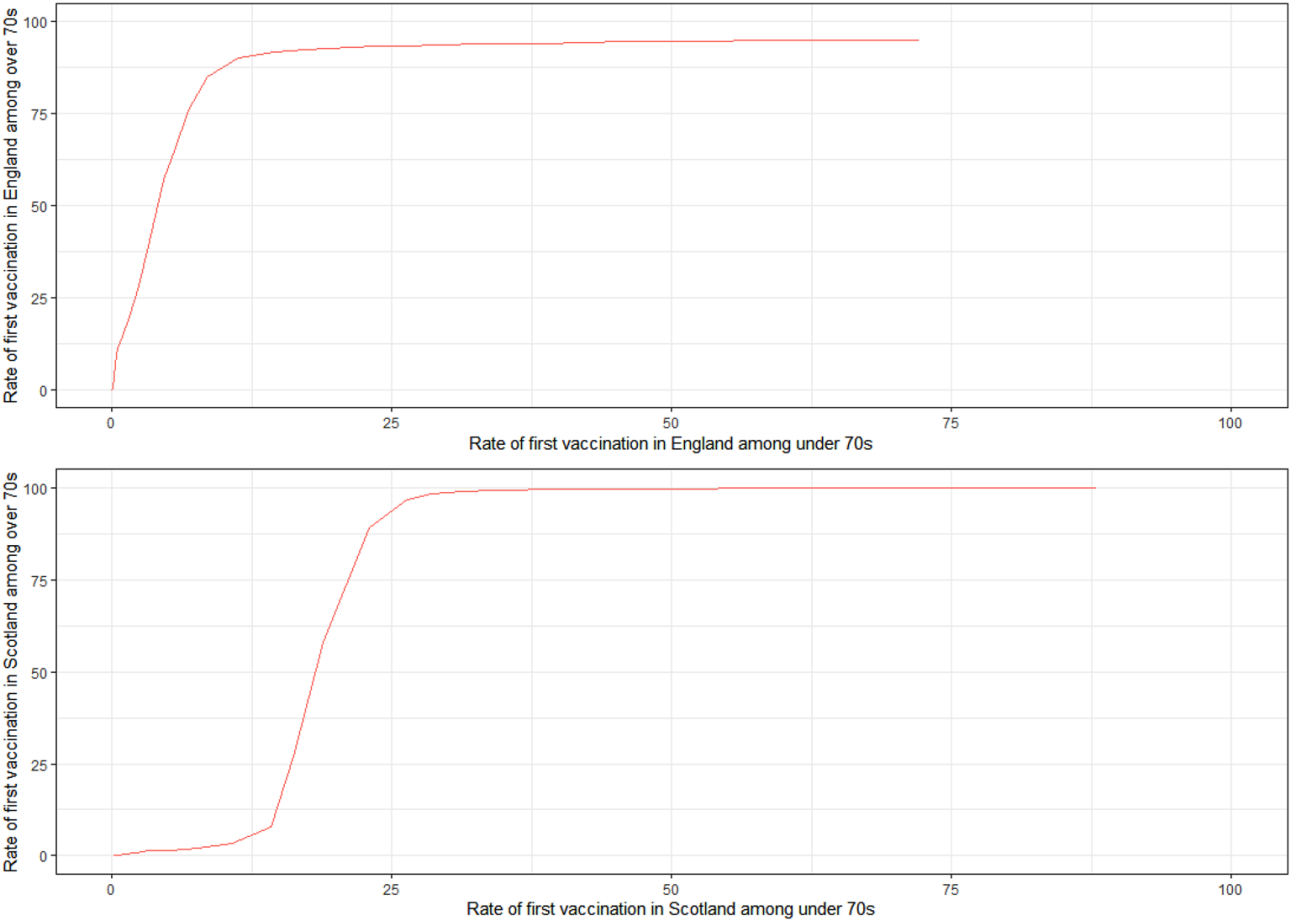
Vaccination rate among over-70s in England and Scotland versus vaccination rates in younger adults (*source*: https://coronavirus.data.gov.uk/details/download). Data were unavailable for Northern Ireland and Wales.

The probability of any physical contacts outside of the household (with under-18s, 18–69 year-olds, and over-70s) increased as population-level vaccination coverage increased overall (Fig. 4A). For all three contact groups, there were distinct increases in the probability of any physical contacts when the population vaccination levels were between 5% and 25%, when the most vulnerable individuals were being vaccinated. This is illustrated in Fig. 5, where the rate of vaccination of over-70s is plotted against the rate of vaccination among the under-70s for the UK as a whole. On average, by the time 10% of under-70s had received a first vaccination, approximately 80% of over-70s had received their first vaccination. For physical contacts with those 18–69 years, the probability of reporting any physical contacts outside of the household in the past 7 days reached a peak of around 0.3, which afterwards stabilized at a probability of approximately 0.2 (Fig. 4A). The probability of any physical contact with over 70s was initially almost zero until 5% of the population received their first vaccination, after which the probability of physical contacts with this age-group started to increase rapidly to 0.1–0.15. Increases were also observed for physical contacts with under-18s when population-level vaccination uptake increased from 0 to 12.5%.

The trends for socially-distanced contacts outside of the household with under-18s and 18–69 year-olds were reasonably flat (Fig. 4B), with the probability of any contacts remaining at around 0.3 and 0.7, respectively. For socially-distanced contacts outside of the household with over 70s, the probability of having any contacts increased steadily with population-level vaccination, peaking at around 0.4 when most eligible individuals were vaccinated.

Increases in population-level vaccination uptake from 0% to 25% were associated with a small gradual increase in the probability of visiting others’ homes (from 0.17 [95% CI 0.16–0.18] to 0.21 [95% CI 0.19–0.22], Fig. 4C). No meaningful difference in the association between the probability of others’ visits to own home and population-level vaccination was observed.

The probability of working outside of the home (note this is restricted to those who reported that they are currently working) increased concurrently with increasing population-level vaccination uptake from around 0.18 [95% CI 0.14–0.21] to 0.62 [95% CI 0.58–0.65] (Fig. 4D). The probability of using public transport for work travel (also restricted to those who reported that they are currently working) in the past 7 days declined concurrently with increasing population-level vaccination uptake 0.43 [95% CI 0.36–0.50] to 0.07 [95% CI 0.05–0.09] (Fig. 4D). A lower probability of taking public transport could be explained by those that increasingly return to outside the home working using private, rather than public, transport.

In interval-censored regressions, a general trend of increasing contacts and household visits was observed across outcomes. It appears to show a behavioural response to population level vaccination where the number of contacts increases as the population vaccination rate increases. Results are presented in Supplementary Figs. 7.

## Discussion

We evaluated how behaviour, measured by the probabilities of physical or socially-distanced contacts with individuals outside the home, visits to others’ homes or of others to one’s own home, work location, and use of public transport, varied in the UK: (i) over time since first vaccination among adults aged 18–64; (ii) over time since the last vulnerable person in the household having their first vaccination among unvaccinated adults aged 18–64 without long-term health conditions; and (iii) by local population-level vaccine uptake in the general population.

We found little to no response in the trends of the measured behaviours to one’s own vaccination, except for a temporary increase in physical contacts likely directly related to getting the vaccination itself. There was limited evidence to suggest that unvaccinated individuals adjusted their behaviour in response to all vulnerable people in their households being vaccinated. There were, however, increases in the population’s probabilities of contacts, in terms of physical contacts, socially-distanced contacts, and working outside the home, as population vaccination rates increased. More specifically, a surge in physical contacts, especially for contacts with those aged 18–69, and to a lesser extent socially-distanced contacts, was reported when most people that were most vulnerable had their first vaccination. Contacts, home visits, and working outside the home increased steadily as vaccination rates in the population increased. This was the case even after controlling for region-specific time trends (Supplementary Fig. 8), which are expected to capture factors such as public health messaging and restrictions which are also related to the outcomes. This suggests risk-compensatory behaviours related to population level vaccination beyond policy changes.

Strengths of this study include the large, nationally-representative, random sample of individuals from the UK. The wide range of behavioural outcomes allows for insights into behavioural change, and internal validity in observing consistent patterns of behaviour where expected. That we observed behaviours under various changing public health mitigation measures is useful for making more general statements about behaviour than would be permissible if we had only observed individuals in strict lockdown or under constant conditions. The data that were collected allow us to control for a set of potentially confounding variables, including household composition, individual characteristics, sociodemographic variables, and time. Finally, being able to measure responses to own, household, and population level vaccination gives greater insight into risk-compensatory behaviours than each alone. Arguably, population level vaccination, where individuals are devoid of control, may be less prone to residual confounding than personal vaccination since individuals do not choose the rates in the areas where they live.

Limitations include that the survey is based on self-reported behaviours. Individuals tend to underreport socially undesirable/stigmatizing behaviours^27^, e.g. having many contacts during periods of strict lockdown, which could translate to reported vaccination behaviours. However, respondents were advised that their answers would be confidential and that the results from the survey would influence the government’s response to the pandemic, the survey was administered by the national statistics agency in the UK, and individuals were paid to participate. These factors lessen underreporting incentives^28–30^. Underreporting would lead to downward bias on estimates; for example, if individuals reported fewer physical contacts than they actually had, then our estimated probability of having these contacts would be lower than the true probability. The fact that only 36% reported having a physical contact in the week they received the vaccination—an increase of 10% compared to the week before the vaccination—suggests that some respondents either thought a vaccination appointment should not be counted as a physical contact or that they perhaps underreported contacts. Given this, we consider these results to be lower bounds of risk-compensatory behaviour. There are other behaviours that were not captured in these outcomes. For example, we did not capture non-work travel (e.g. trips made inter-/intra-regionally). Moreover, we did not account for the risk of the types of interaction in these outcomes (e.g. high risk contact in nightclubs vs low risk attending GP to have a vaccine dose, or high risk contact working in a public-facing role compared to working in a socially-distanced office).

While increasing vaccination levels at the population level may be associated with increased contacts, this may be partly driven by individuals changing their behaviour in line with guidance that has changed as a result of vaccination uptake or based on their own perception of risk for themselves and potentially vulnerable individuals around them. Our analyses cannot distinguish between these two possibilities, although many more factors go into changes to guidance than vaccination alone. We did not explicitly control for the level of infections, nor people’s perception of the level of infections, but did control for this and potential other confounders that may change over time through region-specific splines for calendar time. If there would be almost perfect correlation between calendar time and population-level vaccination uptake, adjustment for calendar time would not be effective in removing confounding by other factors than vaccination uptake simultaneously varying over time. To examine this, we compared models with and without leaving out calendar time or population-level vaccination uptake (supplementary Figs. 14–18 and accompanying Supplementary table 1). The models with only vaccination uptake resulted in markedly different relationship with outcomes than the model with calendar time alone, suggesting that perfect correlation would not likely hinder adjustment for calendar time in our main models. Adjustment for calendar time, as implemented in our main analyses, indeed resulted in different estimated effects of population-level vaccination uptake and a better model fit than models without including this adjustment. However, we cannot rule out unmeasured confounding by factors that are not adequately captured by the region-specific splines for calendar time and other covariates in the model. Finally, due to limited memory in the secure computing environment, it was not possible to estimate models with individual-specific random or fixed effects.

Previous literature about risk compensation associated with SARS-CoV-2 vaccination has been conflicting, with some reporting in favour of behavioural change^20,21^ and others against^19^, but in either case, these were merely conjecture. Our evidence is in favour of a limited degree of risk compensation, though this is related more to population vaccination level, rather than individual level vaccination. These findings suggest a more nuanced role of risk compensation with regard to SARS-CoV-2 vaccination behaviours, and that vaccination itself may not induce behavioural change but rather mass vaccination. Thus, the prior literature could be considered correct or not, depending on the aspect of vaccination-related behaviour that is being referred to. Of course, future studies would be needed to understand behaviours beyond SARS-CoV-2 vaccination, which is a very special case. What these results cannot do is inform behaviours, risk compensatory or otherwise, on vaccination against other respiratory diseases (e.g. influenza).

Physical contacts, socially-distanced contacts, and working outside the home were positively related to population level vaccination. These behaviours are in turn positively related to transmission^31^. This is of concern if individuals are less cautious given evidence of waning immunity^32,33^, especially if vulnerable individuals have not yet received booster vaccinations. Thus, understanding this behaviour may be of further importance as booster vaccinations are administered^34^. Our results, where comparable, are in keeping with the findings of previous studies which find no evidence of higher compliance with guidelines, mask wearing, or social distancing with individual vaccination^23,24^. Our findings provide richer information in terms of more outcomes, but critically also consider risk compensation in respect of vaccination status of potentially frail household members and region-specific vaccination levels. This evidence is likely of use to policymakers at both local and national levels. Having some idea of how individuals’ social behaviours respond to vaccination can help to guide policies. These results have current policy implications for SARS-CoV-2 related policies, such as the behavioural responses to booster vaccinations. These findings may be of value in two possible future settings. One being the case where vaccination-resilient strains of SARS-CoV-2 emerge and further vaccines are required. The second being novel infectious diseases with comparable characteristics to SARS-CoV-2.

## Conclusion

We provide evidence on the behavioural response to COVID-19 vaccination. Patterns of behaviour did not respond to individual nor household vaccinations, but did respond to population-level vaccination. This evidence suggests that population-rate-based risk compensation may have pervaded behaviours in the UK during the SARS-CoV-2 pandemic.

## Methods

### Data

Data are taken from the UK Office for National Statistics Coronavirus (COVID-19) Infection Survey (ISRCTN21086382; https://www.ndm.ox.ac.uk/covid-19/covid-19-infection-survey/protocol-and-information-sheets; details in Pouwels et al.^35^). Individuals were approached from households that were randomly selected from previous surveys and address lists in England, Northern Ireland, Scotland, and Wales to provide a representative sample of the UK population. The survey consists of repeated cross-sectional household surveys with additional serial sampling and longitudinal follow-up. Data collected between 1st October 2020 and 15th September 2021 were used for this analysis. The study received ethical approval from the South Central Berkshire B Research Ethics Committee (20/SC/0195). All methods were performed in accordance with the relevant guidelines and regulations. Informed consent was obtained from all participants. Private households are randomly selected on a continuous basis from address lists and previous surveys to provide a representative sample across the UK. After verbal agreement to participate, a study worker visited each selected household to take written informed consent for individuals aged 2 years and over. Parents or carers provided consent for those aged 2–15 years; those aged 10–15 years also provided written assent. The supplementary materials provide further details of the sampling design.

Data include information on the behavioural outcomes of interest, sociodemographic characteristics, and medical information (https://www.ndm.ox.ac.uk/covid-19/covid-19-infection-survey/case-record-forms for survey questionnaires).

### Vaccination status and population-level uptake

Patients were asked about their vaccination status, including type, number of doses and date(s). Survey participants from England were also linked to administrative records from the National Immunisation Management Service (NIMS)^35^. Where available, we used records from NIMS, otherwise we used records from the survey, since linkage was periodic and NIMS does not contain information about vaccinations received abroad or in Northern Ireland, Scotland, or Wales. Where records were available in both, agreement on type was 98% and dates 95% within ± 7 days.

Data on population level uptake were taken from publicly available government sources (https://coronavirus.data.gov.uk/details/download). This was mapped to the participants’ data at the level of CIS geographical regions In England and Scotland.

### Outcomes

#### 10 behavioural outcomes were self-reported by individuals

(i) The number of physical contacts, e.g. handshake, personal care, including with personal protective equipment, with individuals aged < 18 years old in the past 7 days;
(ii) The number of physical contacts with individuals aged 18–69 years old in the past 7 days;
(iii) The number of physical contacts with individuals aged 70 years and over in the past 7 days;
(iv) The number of socially distanced contacts, worded as, “direct, but not physical, contact”, with individuals aged < 18 years old in the past 7 days;
(v) The number of socially distanced contacts with individuals aged 18–69 years old in the past 7 days;
(vi) The number of socially distanced contacts with individuals aged 70 years and over in the past 7 days;
(vii) The number of times the participant spend one hour or longer inside their own home with someone from another household in the past 7 days;
(viii) The number of times the participant spend time one hour or longer inside the building of another person’s home in the past 7 days;
(ix) Among those that reported engagement in work or studying: mode of travel to work/place of education (grouped as public transport versus other for the current analyses) in the past 7 days; and
(x) Among those that reported engagement in work or studying: main work/study location in the past 7 days.

The physical and socially distanced contact variables (i–vi) were recorded as ordered variables with 5 response categories (0, 1–5, 6–10, 11–20, 21 or more). Both types of home visits (vii and viii) were recorded as ordered variables with 8 response categories (0, 1, 2, 3, 4, 5, 6, 7 times or more). Work or study location was recorded as an ordered variable with three categories (working from home, working from a mix of home and outside of home, working from outside the home).

### Sample eligibility

#### Behaviour changes among vaccinated individuals

Individuals that met the following criteria were included: age 18 years or older, or an individual self-reported a long term health condition (in the UK those with underlying health conditions aged 16 years and over were prioritised after those aged 65 years and over). Preliminary testing plotting difference of the smooths^36^ indicated two groups, patient-facing healthcare workers and over 65s had different behaviours to those under 65 or that did not work in healthcare settings; results are presented in the Supplementary Figs. 1–3. Patient-facing healthcare workers were both prioritised for vaccination and also are expected to have had unusually high contacts during periods of strict mitigation measures given the nature of their job. Older individuals have much higher risk of severe outcomes, generally less frequent contact patterns, and an increasingly small number of unvaccinated older individuals remaining unvaccinated^33^. Individuals were only included if there was evidence that they were vaccinated at any point during the survey to avoid that those not willing to get vaccinated, who are expected to have different behaviours than those that are willing to get vaccinated.

Individuals were included if they had at least one pre-vaccination and one post-vaccination observation between 1st October 2020 and 15th September 2021.

### Influence of vulnerable household member vaccination

This analysis included individuals that lived in a household with a vulnerable individual (defined as either > 65 years or having a long term health condition) but themselves were not considered vulnerable and not yet vaccinated, with visits from the 1st October 2020 and 15th September 2021.

### Influence of population-level uptake

All individuals with visits in the period 1st October 2020 and 15th September 2021 were included in analyses, regardless of vaccination status or reported job.

### Statistical analyses

All analyses used generalized additive ordered categorical regressions that were estimated on the ordinal outcomes^37^. In these models, the linear predictor is a latent variable with estimated thresholds that mark the transitions between levels of the ordered categorical response. For public transport for working travel, which as a binary outcome, a generalized additive logistic regression was used.

Predicted probabilities were estimated for all response categories of the ordinal outcomes. We then collapsed these into binary variables, where the values of the outcomes at zero are treated as zero, and one otherwise (posterior probabilities of being in each category, and their summation to construct the reported probability, are provided in the Supplementary Fig. 14 for physical, outside of household contacts with 18–69 year-olds). In this way, the binary variable captures the margin between “none” and “any”: for contacts, this variable is either no outside of household contacts in the past 7 days (0) or any outside of household contacts in the past 7 days (1); for home visits it is either no visits in the past 7 days (0) or any home visits in the past 7 days (1); for work location, this is either working at home every day in the past 7 days (0) or working in outside of home location (i.e. office, café, etc.) one or more times in the past 7 days (1); and for mode of travel to work, this is either private transport in the past 7 days (0) or public transport (1). Using the ordinal variables in models allows us to exploit all of the information in the data, and collapsing them into posterior binary variables eases exposition, aligns the results across outcomes with different numbers of categories, and focusses the analyses on the margin of “none” and “any” which is most useful for understanding transmission.

### Behaviour changes among vaccinated individuals

This analysis examined the behavioural response to individual vaccination. Outcomes were modelled from 180 days prior to first vaccination to 120 after the first vaccination (given few visits outside of this range). To capture non-linear behavioural change, we used thin plate splines on time from first vaccination (the number of basis functions (*k* in the mgcv package^38^) was the number of unique values divided by 3). The degree of smoothing of the splines was optimised using a fast implementation of restricted maximum likelihood (REML)^38,39^. Regressions controlled for confounding with individual-specific characteristics of gender, ethnicity, socioeconomic status (rank of index of multiple deprivation calculated separately for each country in the United Kingdom^40–43^), urban/rural classification^11,12,44^, the stage of lockdown (and corresponding restrictions applied), household size, whether the household is multigenerational (household individuals aged 16 or younger and individuals aged school year 12 to age 49 and individuals aged 50 +), and if the individual ever reported a long-term healthcare condition. Calendar time by region/country (9 regions in England and Northern Ireland, Scotland, and Wales) interactions were captured using thin plate splines (the number of basis functions was the number of unique values divided by 3) interacting with region/country (factor smooth interaction) to control for non-linear confounding of region/country-specific behavioural change over time (see Supplementary Fig. 8 for an example of physical contacts with under 18 year-olds). Thin plate splines were also applied to age to control for non-linear confounding of age-specific behaviour.

Testing changes in behavioural responses to individual vaccination involved testing trends pre- and post-vaccination. More specifically, the gradient in the outcomes (over time from vaccination) in a period before vaccination (− 50 days to − 14 days) was tested against two periods (1–13 days and 14–50 days) after vaccination, arguing that longer-term changes were less likely related to vaccination. The period 50 days to 14 days was used as the comparator to avoid conflating any changed post-vaccination behaviours with any behavioural shifts leading up to having a vaccine. This was conducted using 10,000 simulations based on pseudo-random draws from the posterior distributions of the fitted GAM models using the Gaussian approximation to the posterior of the model coefficients. The pseudo-random draws are obtained from a multivariate normal with mean vector equal to the estimated model coefficients and covariance matrix equal to the covariance matrix of the coefficients. For each draw, the gradient of the outcome was computed for the pre- and the two post-periods; and the differences in the gradients between the pre-period and each of the post-periods, respectively, were taken. Across the draws, the median, 2.5th, and 97.5th percentiles of the distribution of differences were taken and compared to assess if the gradients of the outcomes changed between pre- and post-vaccination periods.

### Influence of vulnerable household member vaccination

This analysis examined the behavioural response to vaccination of vulnerable household members as described above. This analysis was similar to that for vaccinated individuals, except the definition of vaccination date was altered to be the date of vaccination for the last vulnerable individual in the household to be vaccinated. In addition, this analysis was limited to the subset of (a) individuals considered to be non-vulnerable and (c) observations until individuals themselves received a first vaccination. To capture non-linear behavioural change, we used thin plate splines on time from first household vaccination^45^ (the number of basis functions (*k* in the mgcv package^38^) was based on the number of unique values divided by 3). Model specification was otherwise identical to the analysis of vaccinated individuals.

### Influence of population-level uptake

This analysis examined the behavioural response to vaccination rates at the population level rather than to individual vaccination. In these models, the time from first vaccination variable was replaced with the percentage of individuals in the population that had been vaccinated. Daily rates of vaccine uptake were available for local areas and these were merged to the CIS data by CIS geographical area to enable modelling. To capture non-linear behavioural change, we used thin plate splines on population-level-vaccination^45^ (the number of basis functions (*k* in the mgcv package^38^) was the number of unique values divided by 3). In addition, a variable for whether individuals were patient-facing healthcare workers was included. Thin plate splines were also applied to age to control for non-linear confounding of age-specific behaviour (where again the number of basis functions was the number of unique values divided by 3); age was truncated at 95 to reduce the influence of outliers. A categorical variable was defined which indicated if the individual was a child, a vaccinated adult or an unvaccinated adult. This variable was interacted with the thin-plate splines on population level vaccination to allow for differential behavioural responses for each of the groups.

### Specification and sensitivity analyses

The proportional odds assumption was tested in preliminary analyses by interacting the threshold parameters with a post-vaccination dummy variable. No evidence of variation in the response over the range of the outcomes was found. We tested tensor splines for time-age-region interactions (knots divided the time range at 3-day intervals and the age range at 3-year intervals) to control for non-linear confounding of age- and region-specific behavioural change over time. There was no improvement in model fit nor impact on the difference in the outcomes over time. Thus, the simpler specification, i.e. region by time interaction, was retained.

Models examined if behaviours were consistent in three groups (relative to the remaining sample): those over age 65, reporting working in patient-facing healthcare, and reporting a long-term health condition. Evidence of different patterns of behaviour were found for those over 65 years and for patient-facing healthcare workers, but not for those self-reporting long-term health conditions (results are presented in the Supplementary Figs. 1–3). Differential behavioural trends in response to ChAdOx1 versus BNT162b2 vaccines were evaluated by plotting difference of the smooths (other types of vaccine, e.g. mRNA-1273 (Moderna), were controlled for separately). No differences were observed in behaviours for ChAdOx1 versus the BNT162b2 vaccines (Supplementary Fig. 9). For the population-level vaccination models, including an interaction between the thin-plate splines for time-from-vaccination and a categorical variable indicating if the individual was a child, a vaccinated adult or an unvaccinated adult improved the fit of the model and was therefore used. A model that included both time from own vaccination and population level vaccination were estimated to ensure that the results were robust.

Models that use the date of second, rather than first, vaccination yielded similar patterns across the outcomes. Results are shown in Supplementary Figs. 10–12. In addition, we analysed responses to population level vaccination among those that had been vaccinated and consistent with the eligibility criteria described for the analysis of own vaccination. Results are presented in Supplementary Fig. 13. In contrast to the full sample, an initial peak in the probability of contacts in the first 25% of population vaccination is not observed, suggesting this peak is driven by children and the unvaccinated; otherwise the patterns of behaviour are consistent. Interval regressions were estimated to give complementary results on associations between vaccination and the number of contacts over time. These models were implemented using the ‘survival’ package and used p-splines—with the degrees of freedom based on the AIC—to allow for non-linear effects of the exposures of interest.

To evaluate whether passage of time—potentially reflective of pandemic fatigue—or increases in vaccination rates are more important for behavioural responses, we added a comparison between models with calendar time only, models with population-level vaccination uptake only, and models with both calendar time and vaccination (supplementary Figs. 14–16).

To assess whether almost perfect correlation between vaccination uptake and time when modelling both using flexible splines would hinder our inference, we also explored the use of calendar time measured in weeks (by 1 and 2 weeks), as one would expect pandemic fatigue to be rather stable within 1- or 2-week intervals while population-level vaccination uptake changed quickly within these intervals (e.g. 5% increase in the first week of February 2021).The results appear to be robust to the specification of calendar time. Results for the weekly and fortnightly models are presented in supplementary Figs. 17 and 18.

## Supplementary Information

Supplementary Information.

## Data Availability

Data are still being collected for the COVID-19 Infection Survey. De-identified study data are available for access by accredited researchers in the ONS Secure Research Service (SRS) for accredited research purposes under part 5, chapter 5 of the Digital Economy Act 2017. For further information about accreditation, contact Research.Support@ons.gov.uk or visit the SRS website.

## Code availability

All statistical analyses were performed using standard functions in the following R packages: ggplot2 (version 3.3.2), mgcv (version 1.8–40), survival (version 3.3–1), dplyr (version 1.0.2), , haven (version 2.3.1), sandwich (version 3.0–0), ggeffects (version 1.0.1), . Analysis code for the BAM models, predictions, and gradients is available at: https://github.com/johnbuckell/COVID-19-vaccination-risk-compensatory-behaviours-and-contacts-in-the-UK.
