# Supplementary material for: COVID-19 vaccination, risk-compensatory behaviours, and contacts in the UK
Source: Sci Rep. 2023 May 25;13:8441. doi: 10.1038/s41598-023-34244-2 (PMC10209557; doi:10.1038/s41598-023-34244-2)
Supplement: Supplementary file 1 — Supplementary Information. [file 41598_2023_34244_MOESM1_ESM.docx]

**Age, long term health conditions, and health and social care workers testing**


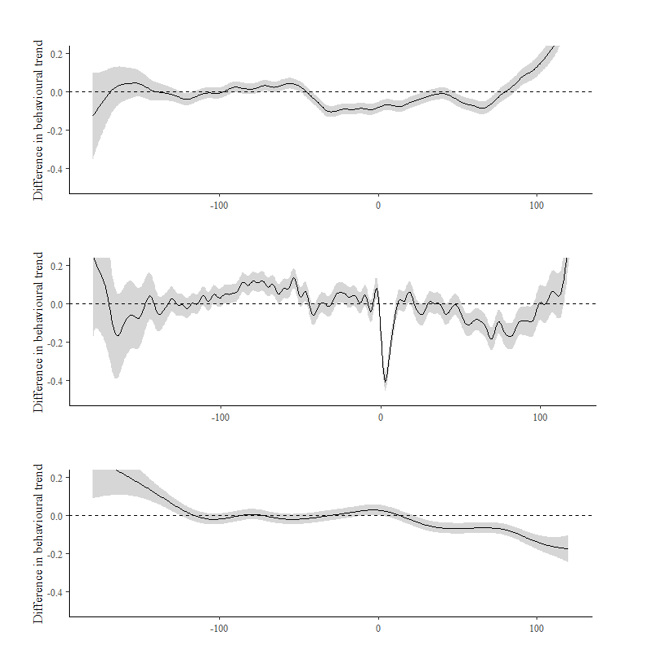


Supplementary figure 1: Differences in smooths for age>65 (vs. age<=65). Top panel: others in own home in the past 7 days. Middle panel: physical contacts with 18 to 69 year-olds in the past 7 days. Bottom panel: Socially-distanced contacts with 18 to 69 year-olds in the past 7 days. Evidence to suggest behavioural trends diverge between ages over and under 65, hence main analyses focussed on those aged 18-64.


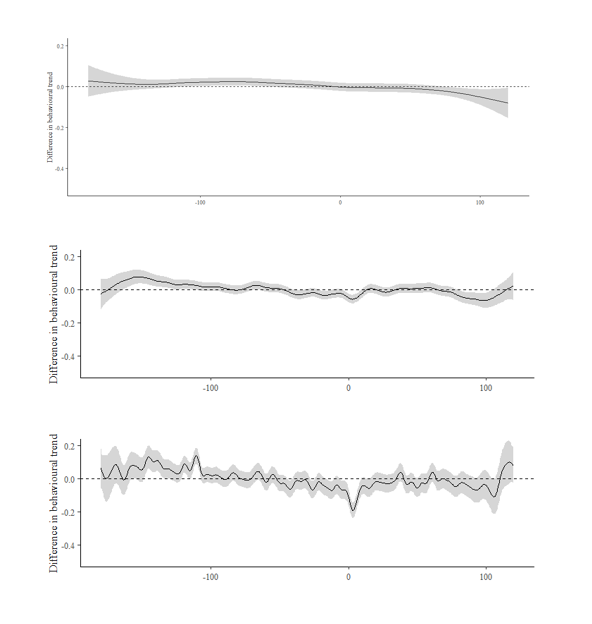


Supplementary figure 2: Differences in smooths for individuals with long-term health conditions (vs. individuals without long-term health conditions). Top panel: others in own home in the past 7 days. Middle panel: physical contacts with 18 to 69 year-olds in the past 7 days. Bottom panel: Socially-distanced contacts with 18 to 69 year-olds in the past 7 days. No evidence to suggest behavioural trends diverge between individuals with and without long-term health conditions.


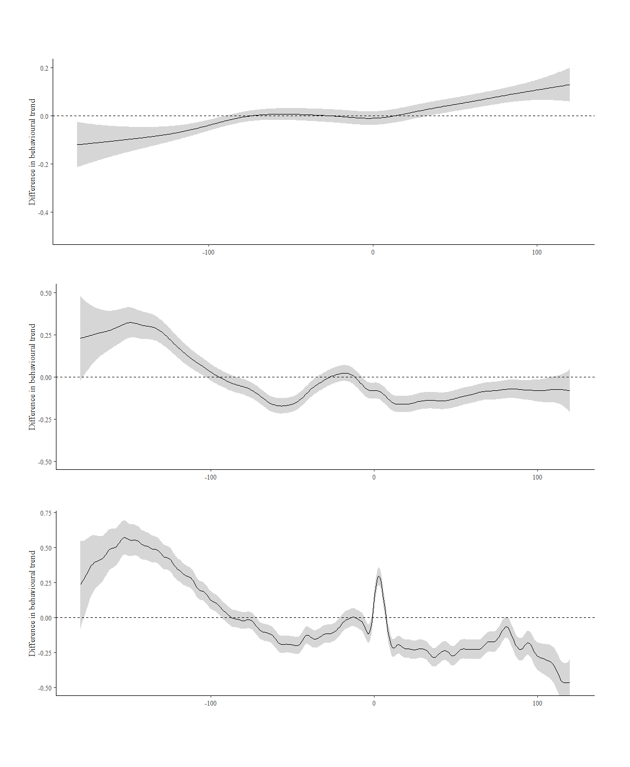


Supplementary figure 3: Differences in smooths for patient-facing healthcare workers (vs. non-health and social care workers). Top panel: others in own home in the past 7 days. Middle panel: physical contacts with 18 to 69 year-olds in the past 7 days. Bottom panel: Socially-distanced contacts with 18 to 69 year-olds in the past 7 days. Evidence to suggest behavioural trends diverge between individuals who are and are not health and social care workers.

Distribution of vaccine take up over time, by age group


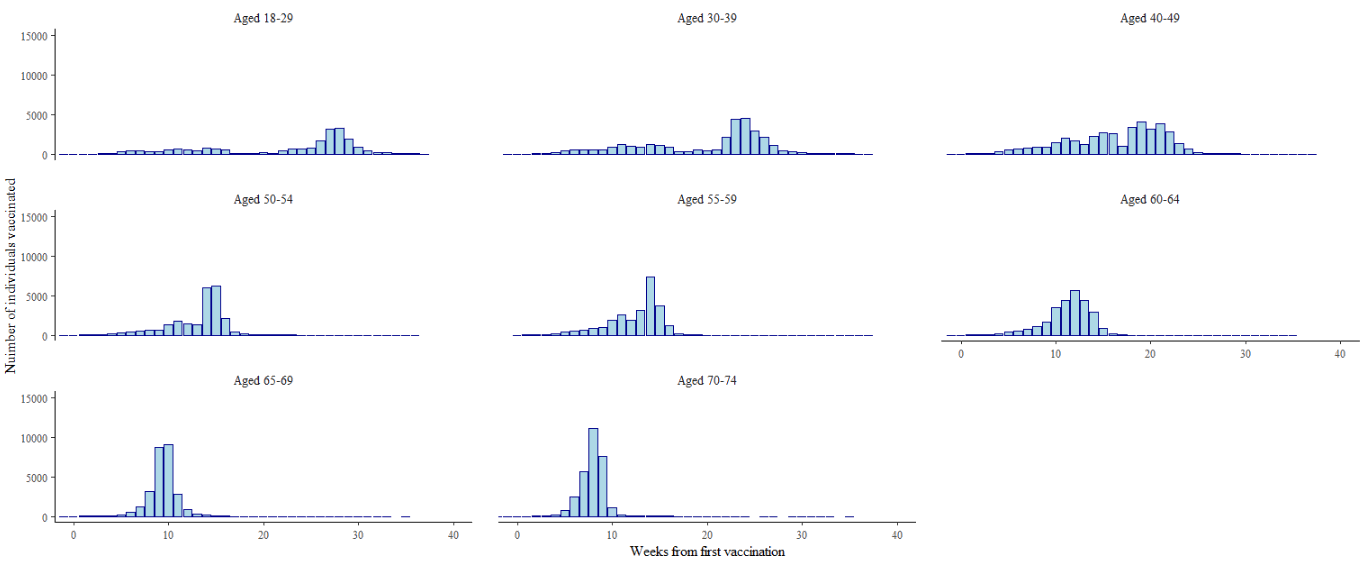


Supplementary figure 4: weekly counts of vaccination uptake by age group

**Behavioural response to first vaccination using interval regressions**


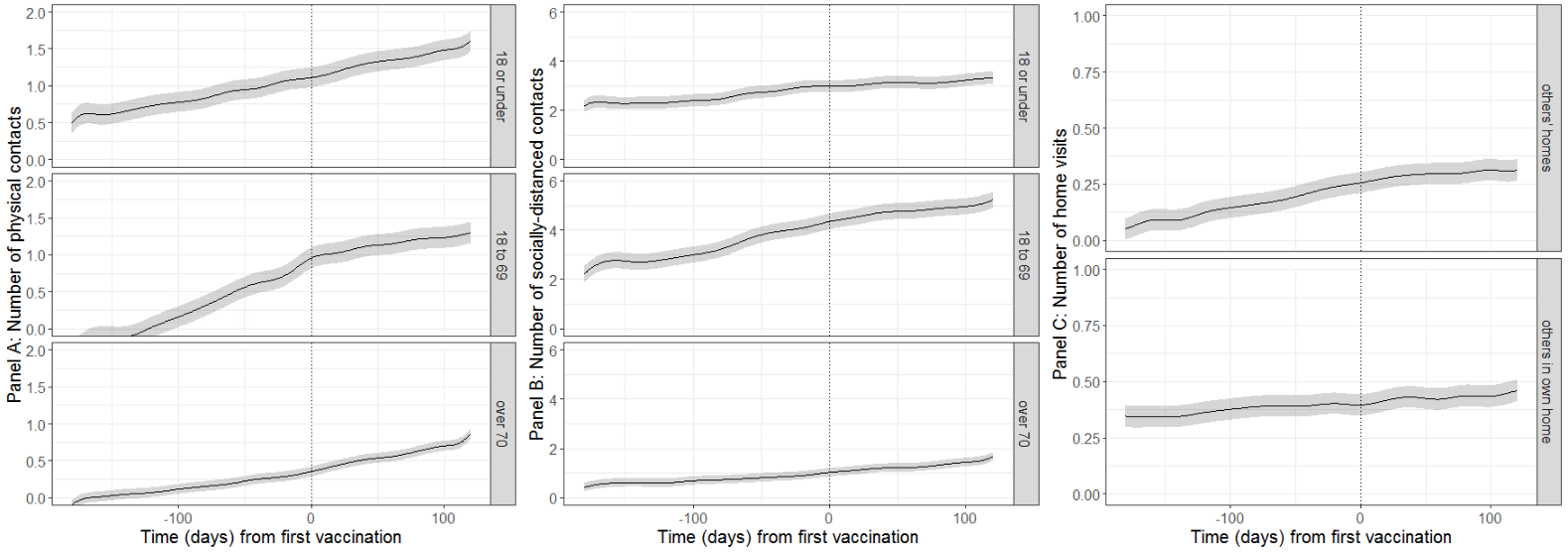


Supplementary figure 5: Predicted counts of behavioural outcomes for individuals aged 18-64y by time from first vaccination, first dose. Left panel: past 7-day reported physical, outside of household contacts; Middle panel: past 7-day reported socially-distanced, outside of household contacts; Right panel: past 7-day reported home visits. Dotted line shows day of own first vaccination. “18 or under”, “18 to 69” and “over 70” denote the ages of the people with whom individuals in the sample had contact.


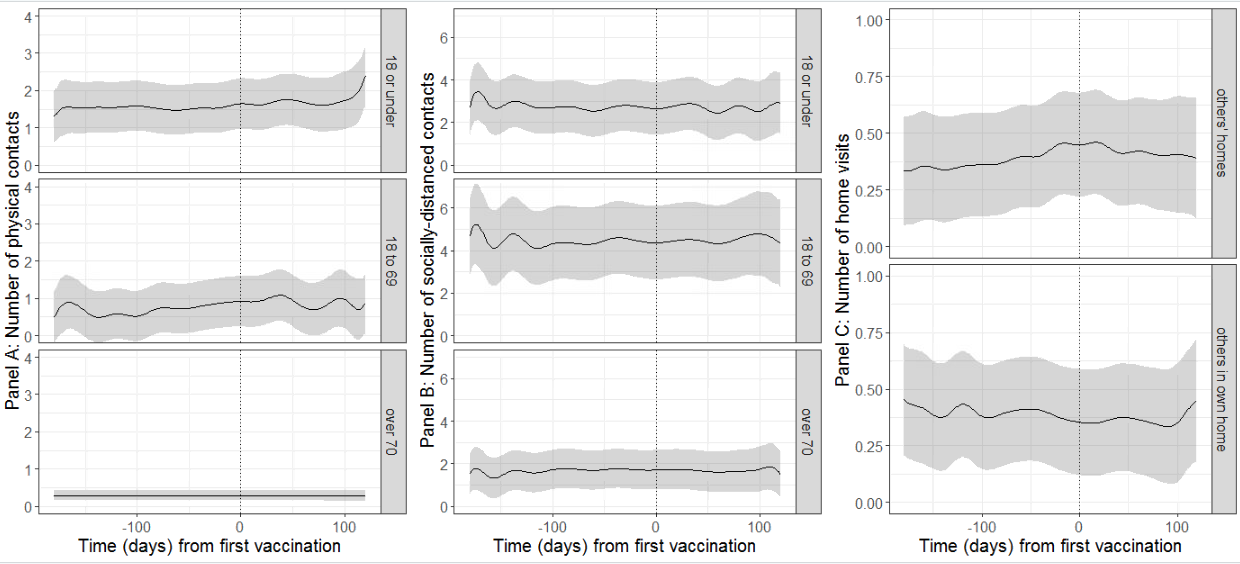


Supplementary figure 6: Predicted counts of unvaccinated individuals aged 18-64y by time to the first day that all vulnerable household members are vaccinated. Left panel: past 7-day reported physical, outside of household contacts; Middle panel: past 7-day reported socially-distanced, outside of household contacts; Right panel: past 7-day reported home visits. Dotted line shows day of own first vaccination. “18 or under”, “18 to 69” and “over 70” denote the ages of the people with whom individuals in the sample had contact.


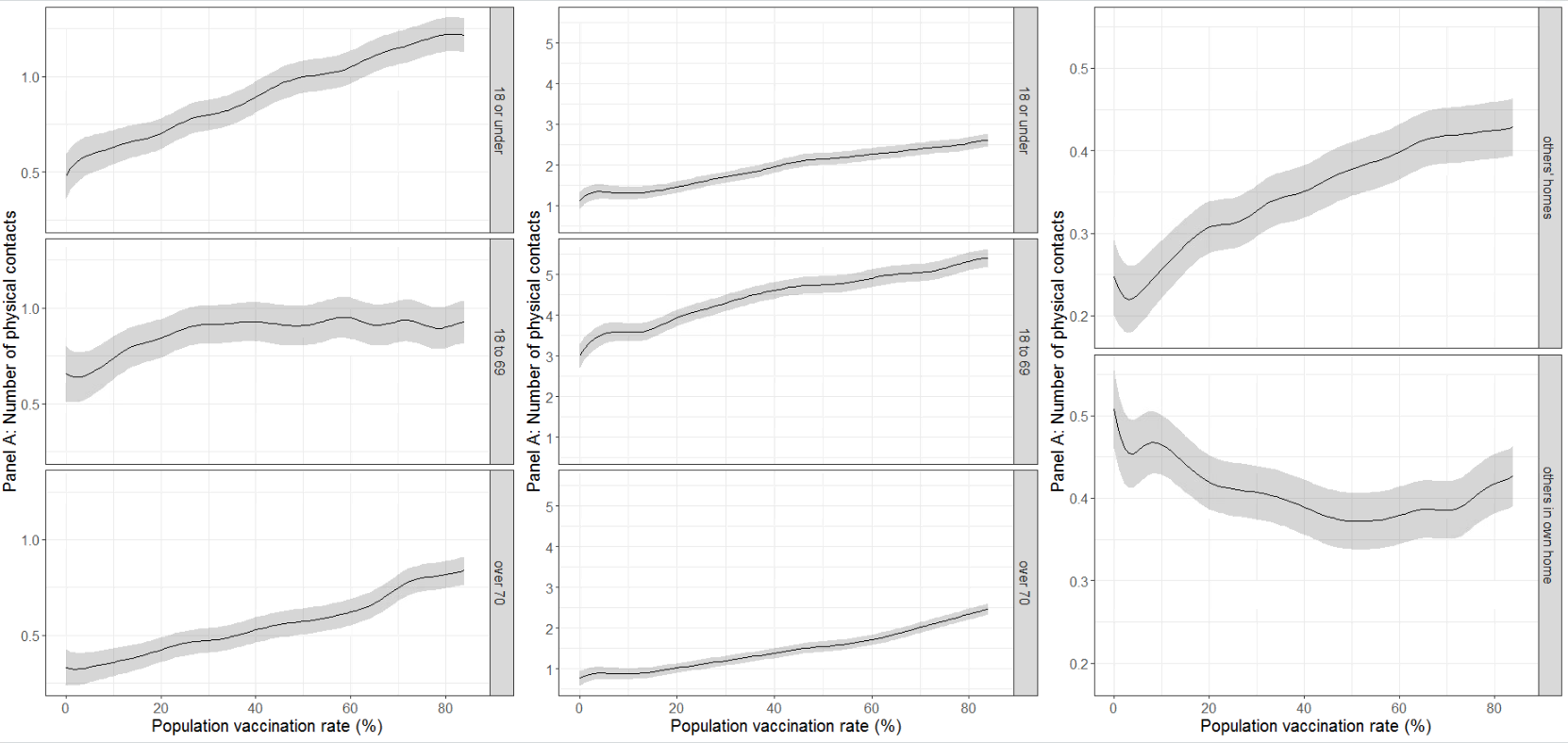


Supplementary figure 7: Predicted counts for all individuals as the population level vaccination increased. Left panel: past 7-day reported physical, outside of household contacts; Middle panel: past 7-day reported socially-distanced, outside of household contacts; Right panel: past 7-day reported home visits. Dotted line shows day of own first vaccination. “18 or under”, “18 to 69” and “over 70” denote the ages of the people with whom individuals in the sample had contact.

Supplementary figure **8:Calendar time by region/country (9 regions in England and Northern Ireland, Scotland, and Wales) for physical contacts with under 18 year-olds; vaccinated individuals**


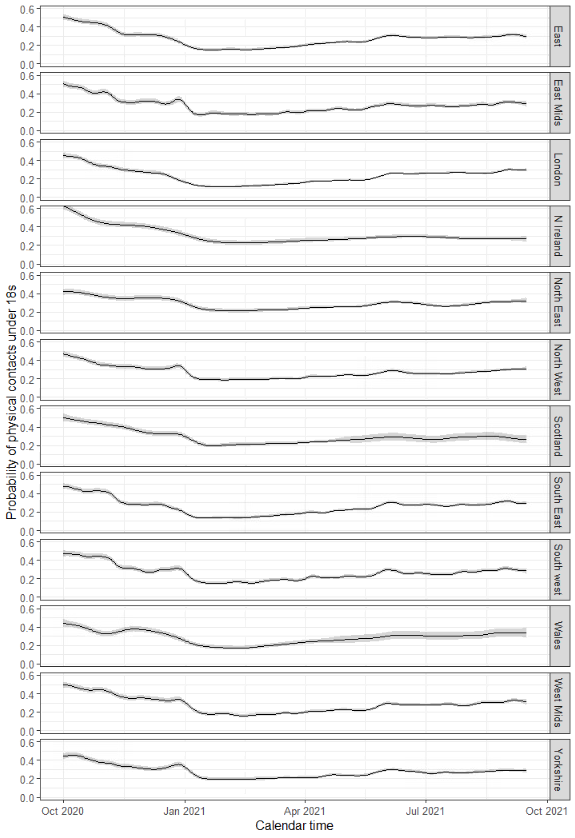


**Sampling design**

The following information on the sampling design can also be found at: <https://www.ons.gov.uk/peoplepopulationandcommunity/healthandsocialcare/conditionsanddiseases/methodologies/covid19infectionsurveypilotmethodsandfurtherinformation#study-design-sampling>

The Office for National Statistics (ONS) COVID-19 Infection Survey randomly selects private households on a continuous basis from address lists and previous surveys to provide a representative sample across the UK (England, Wales, Northern Ireland, and Scotland). At the start of the study, all respondents were individuals who have previously participated in an ONS social survey. To take part, invited households opted into the survey by contacting IQVIA to arrange a household visit. Since the end of July 2020, we further expanded the survey to invite a random sample of households from AddressBase, which is a commercially available list of addresses maintained by the Ordnance Survey.

Response rates varied across the four countries and depending on the period of data collection and were related to the scale at which information was collected. In England, response rates were 51% from April 26^th^ 2020, with 10,332/20,276 households registered of those invited; 43% from 31^st^ May 2020, with 39,542/91,146 households registered of those invited; and 13% from 13^th^ July 2020, with 177,923/1,400,783 households registered of those invited. In Wales, response rates were 41% from June 29^th^ 2020, with 7,090/17,328 households registered of those invited; and 14% from October 5^th^ 2020, with 7,256/51,528 households registered of those invited. In Northern Ireland, response rates were 44% from July 26^th^ 2020, with 7,421/16,977 households registered of those invited. In Scotland, response rates were 13% from September 14^th^ 2020, with 23,941/184,939 households registered of those invited. For full details, see <https://www.ons.gov.uk/peoplepopulationandcommunity/healthandsocialcare/conditionsanddiseases/datasets/covid19infectionsurveytechnicaldata>

Attrition rates are not available and also difficult to determine in near real-time for such a large-scale study as e.g. someone might not be present at one or more visits might still be in the survey and be present at a next household visit. Considering this attrition rates are very low, particularly given the generous compensation participants receive for each visit (vouchers worth 25 GBP).

**Prediction for levels of ordered variable and construction of probability of any 18 to 69 year old, outside of household, physical contacts**

| Time from vaccination | fit.1 | fit.2 | fit.3 | fit.4 | fit.5 | Probability of some contacts |
| --- | --- | --- | --- | --- | --- | --- |
| -180 | 0.871 | 0.111 | 0.010 | 0.004 | 0.004 | 0.129 |
| -179 | 0.869 | 0.113 | 0.010 | 0.004 | 0.004 | 0.131 |
| -178 | 0.867 | 0.115 | 0.010 | 0.004 | 0.004 | 0.133 |
| -177 | 0.865 | 0.116 | 0.010 | 0.004 | 0.004 | 0.135 |
| -176 | 0.864 | 0.118 | 0.011 | 0.004 | 0.004 | 0.136 |
| -175 | 0.863 | 0.119 | 0.011 | 0.004 | 0.004 | 0.137 |
| -174 | 0.862 | 0.119 | 0.011 | 0.004 | 0.004 | 0.138 |
| -173 | 0.861 | 0.120 | 0.011 | 0.004 | 0.004 | 0.139 |
| -172 | 0.861 | 0.120 | 0.011 | 0.004 | 0.004 | 0.139 |
| -171 | 0.861 | 0.120 | 0.011 | 0.004 | 0.004 | 0.139 |
| -170 | 0.860 | 0.121 | 0.011 | 0.004 | 0.004 | 0.140 |
| -169 | 0.860 | 0.121 | 0.011 | 0.004 | 0.004 | 0.140 |
| -168 | 0.860 | 0.121 | 0.011 | 0.004 | 0.004 | 0.140 |
| -167 | 0.859 | 0.122 | 0.011 | 0.004 | 0.004 | 0.141 |
| -166 | 0.859 | 0.122 | 0.011 | 0.004 | 0.004 | 0.141 |
| -165 | 0.858 | 0.122 | 0.011 | 0.004 | 0.004 | 0.142 |
| -164 | 0.858 | 0.122 | 0.011 | 0.004 | 0.004 | 0.142 |
| -163 | 0.858 | 0.122 | 0.011 | 0.004 | 0.004 | 0.142 |
| -162 | 0.858 | 0.122 | 0.011 | 0.004 | 0.004 | 0.142 |
| -161 | 0.859 | 0.122 | 0.011 | 0.004 | 0.004 | 0.141 |
| -160 | 0.859 | 0.122 | 0.011 | 0.004 | 0.004 | 0.141 |
| -159 | 0.859 | 0.122 | 0.011 | 0.004 | 0.004 | 0.141 |
| -158 | 0.858 | 0.122 | 0.011 | 0.004 | 0.004 | 0.142 |
| -157 | 0.858 | 0.123 | 0.011 | 0.004 | 0.004 | 0.142 |
| -156 | 0.858 | 0.123 | 0.011 | 0.004 | 0.004 | 0.142 |
| -155 | 0.858 | 0.123 | 0.011 | 0.004 | 0.004 | 0.142 |
| -154 | 0.857 | 0.123 | 0.011 | 0.004 | 0.004 | 0.143 |
| -153 | 0.858 | 0.123 | 0.011 | 0.004 | 0.004 | 0.142 |
| -152 | 0.858 | 0.123 | 0.011 | 0.004 | 0.004 | 0.142 |
| -151 | 0.859 | 0.122 | 0.011 | 0.004 | 0.004 | 0.141 |
| -150 | 0.859 | 0.122 | 0.011 | 0.004 | 0.004 | 0.141 |
| -149 | 0.859 | 0.122 | 0.011 | 0.004 | 0.004 | 0.141 |
| -148 | 0.859 | 0.122 | 0.011 | 0.004 | 0.004 | 0.141 |
| -147 | 0.859 | 0.122 | 0.011 | 0.004 | 0.004 | 0.141 |
| -146 | 0.858 | 0.122 | 0.011 | 0.004 | 0.004 | 0.142 |
| -145 | 0.858 | 0.122 | 0.011 | 0.004 | 0.004 | 0.142 |
| -144 | 0.859 | 0.122 | 0.011 | 0.004 | 0.004 | 0.141 |
| -143 | 0.859 | 0.121 | 0.011 | 0.004 | 0.004 | 0.141 |
| -142 | 0.860 | 0.121 | 0.011 | 0.004 | 0.004 | 0.140 |
| -141 | 0.860 | 0.121 | 0.011 | 0.004 | 0.004 | 0.140 |
| -140 | 0.859 | 0.122 | 0.011 | 0.004 | 0.004 | 0.141 |
| -139 | 0.859 | 0.122 | 0.011 | 0.004 | 0.004 | 0.141 |
| -138 | 0.858 | 0.122 | 0.011 | 0.004 | 0.004 | 0.142 |
| -137 | 0.858 | 0.122 | 0.011 | 0.004 | 0.004 | 0.142 |
| -136 | 0.858 | 0.122 | 0.011 | 0.004 | 0.004 | 0.142 |
| -135 | 0.859 | 0.122 | 0.011 | 0.004 | 0.004 | 0.141 |
| -134 | 0.859 | 0.122 | 0.011 | 0.004 | 0.004 | 0.141 |
| -133 | 0.859 | 0.122 | 0.011 | 0.004 | 0.004 | 0.141 |
| -132 | 0.859 | 0.122 | 0.011 | 0.004 | 0.004 | 0.141 |
| -131 | 0.859 | 0.122 | 0.011 | 0.004 | 0.004 | 0.141 |
| -130 | 0.858 | 0.122 | 0.011 | 0.004 | 0.004 | 0.142 |
| -129 | 0.858 | 0.123 | 0.011 | 0.004 | 0.004 | 0.142 |
| -128 | 0.857 | 0.123 | 0.011 | 0.004 | 0.004 | 0.143 |
| -127 | 0.857 | 0.124 | 0.011 | 0.004 | 0.004 | 0.143 |
| -126 | 0.856 | 0.124 | 0.011 | 0.004 | 0.004 | 0.144 |
| -125 | 0.856 | 0.124 | 0.011 | 0.004 | 0.004 | 0.144 |
| -124 | 0.857 | 0.124 | 0.011 | 0.004 | 0.004 | 0.143 |
| -123 | 0.857 | 0.123 | 0.011 | 0.004 | 0.004 | 0.143 |
| -122 | 0.858 | 0.123 | 0.011 | 0.004 | 0.004 | 0.142 |
| -121 | 0.858 | 0.123 | 0.011 | 0.004 | 0.004 | 0.142 |
| -120 | 0.857 | 0.123 | 0.011 | 0.004 | 0.004 | 0.143 |
| -119 | 0.857 | 0.124 | 0.011 | 0.004 | 0.004 | 0.143 |
| -118 | 0.856 | 0.124 | 0.011 | 0.004 | 0.004 | 0.144 |
| -117 | 0.855 | 0.125 | 0.011 | 0.004 | 0.004 | 0.145 |
| -116 | 0.854 | 0.126 | 0.011 | 0.004 | 0.004 | 0.146 |
| -115 | 0.853 | 0.126 | 0.011 | 0.004 | 0.004 | 0.147 |
| -114 | 0.852 | 0.127 | 0.012 | 0.004 | 0.004 | 0.148 |
| -113 | 0.851 | 0.128 | 0.012 | 0.004 | 0.005 | 0.149 |
| -112 | 0.850 | 0.129 | 0.012 | 0.004 | 0.005 | 0.150 |
| -111 | 0.849 | 0.130 | 0.012 | 0.004 | 0.005 | 0.151 |
| -110 | 0.849 | 0.130 | 0.012 | 0.004 | 0.005 | 0.151 |
| -109 | 0.849 | 0.131 | 0.012 | 0.004 | 0.005 | 0.151 |
| -108 | 0.848 | 0.131 | 0.012 | 0.004 | 0.005 | 0.152 |
| -107 | 0.848 | 0.131 | 0.012 | 0.004 | 0.005 | 0.152 |
| -106 | 0.848 | 0.131 | 0.012 | 0.004 | 0.005 | 0.152 |
| -105 | 0.847 | 0.132 | 0.012 | 0.004 | 0.005 | 0.153 |
| -104 | 0.847 | 0.132 | 0.012 | 0.004 | 0.005 | 0.153 |
| -103 | 0.847 | 0.132 | 0.012 | 0.004 | 0.005 | 0.153 |
| -102 | 0.847 | 0.132 | 0.012 | 0.004 | 0.005 | 0.153 |
| -101 | 0.847 | 0.132 | 0.012 | 0.004 | 0.005 | 0.153 |
| -100 | 0.847 | 0.132 | 0.012 | 0.004 | 0.005 | 0.153 |
| -99 | 0.847 | 0.132 | 0.012 | 0.004 | 0.005 | 0.153 |
| -98 | 0.846 | 0.132 | 0.012 | 0.004 | 0.005 | 0.154 |
| -97 | 0.846 | 0.133 | 0.012 | 0.004 | 0.005 | 0.154 |
| -96 | 0.845 | 0.133 | 0.012 | 0.004 | 0.005 | 0.155 |
| -95 | 0.845 | 0.134 | 0.012 | 0.004 | 0.005 | 0.155 |
| -94 | 0.844 | 0.134 | 0.012 | 0.004 | 0.005 | 0.156 |
| -93 | 0.844 | 0.135 | 0.012 | 0.004 | 0.005 | 0.156 |
| -92 | 0.843 | 0.135 | 0.012 | 0.004 | 0.005 | 0.157 |
| -91 | 0.843 | 0.135 | 0.012 | 0.004 | 0.005 | 0.157 |
| -90 | 0.843 | 0.135 | 0.012 | 0.004 | 0.005 | 0.157 |
| -89 | 0.843 | 0.135 | 0.012 | 0.004 | 0.005 | 0.157 |
| -88 | 0.843 | 0.135 | 0.012 | 0.005 | 0.005 | 0.157 |
| -87 | 0.842 | 0.136 | 0.012 | 0.005 | 0.005 | 0.158 |
| -86 | 0.842 | 0.136 | 0.013 | 0.005 | 0.005 | 0.158 |
| -85 | 0.841 | 0.137 | 0.013 | 0.005 | 0.005 | 0.159 |
| -84 | 0.840 | 0.137 | 0.013 | 0.005 | 0.005 | 0.160 |
| -83 | 0.840 | 0.138 | 0.013 | 0.005 | 0.005 | 0.160 |
| -82 | 0.839 | 0.138 | 0.013 | 0.005 | 0.005 | 0.161 |
| -81 | 0.839 | 0.139 | 0.013 | 0.005 | 0.005 | 0.161 |
| -80 | 0.838 | 0.139 | 0.013 | 0.005 | 0.005 | 0.162 |
| -79 | 0.838 | 0.140 | 0.013 | 0.005 | 0.005 | 0.162 |
| -78 | 0.837 | 0.140 | 0.013 | 0.005 | 0.005 | 0.163 |
| -77 | 0.837 | 0.141 | 0.013 | 0.005 | 0.005 | 0.163 |
| -76 | 0.836 | 0.141 | 0.013 | 0.005 | 0.005 | 0.164 |
| -75 | 0.835 | 0.142 | 0.013 | 0.005 | 0.005 | 0.165 |
| -74 | 0.835 | 0.142 | 0.013 | 0.005 | 0.005 | 0.165 |
| -73 | 0.835 | 0.142 | 0.013 | 0.005 | 0.005 | 0.165 |
| -72 | 0.835 | 0.142 | 0.013 | 0.005 | 0.005 | 0.165 |
| -71 | 0.836 | 0.142 | 0.013 | 0.005 | 0.005 | 0.164 |
| -70 | 0.836 | 0.141 | 0.013 | 0.005 | 0.005 | 0.164 |
| -69 | 0.837 | 0.141 | 0.013 | 0.005 | 0.005 | 0.163 |
| -68 | 0.836 | 0.141 | 0.013 | 0.005 | 0.005 | 0.164 |
| -67 | 0.836 | 0.141 | 0.013 | 0.005 | 0.005 | 0.164 |
| -66 | 0.835 | 0.142 | 0.013 | 0.005 | 0.005 | 0.165 |
| -65 | 0.834 | 0.143 | 0.013 | 0.005 | 0.005 | 0.166 |
| -64 | 0.833 | 0.144 | 0.013 | 0.005 | 0.005 | 0.167 |
| -63 | 0.832 | 0.145 | 0.013 | 0.005 | 0.005 | 0.168 |
| -62 | 0.831 | 0.146 | 0.014 | 0.005 | 0.005 | 0.169 |
| -61 | 0.829 | 0.147 | 0.014 | 0.005 | 0.005 | 0.171 |
| -60 | 0.828 | 0.147 | 0.014 | 0.005 | 0.005 | 0.172 |
| -59 | 0.828 | 0.148 | 0.014 | 0.005 | 0.005 | 0.172 |
| -58 | 0.827 | 0.149 | 0.014 | 0.005 | 0.005 | 0.173 |
| -57 | 0.826 | 0.149 | 0.014 | 0.005 | 0.005 | 0.174 |
| -56 | 0.826 | 0.150 | 0.014 | 0.005 | 0.005 | 0.174 |
| -55 | 0.826 | 0.150 | 0.014 | 0.005 | 0.005 | 0.174 |
| -54 | 0.826 | 0.150 | 0.014 | 0.005 | 0.005 | 0.174 |
| -53 | 0.826 | 0.150 | 0.014 | 0.005 | 0.005 | 0.174 |
| -52 | 0.826 | 0.150 | 0.014 | 0.005 | 0.005 | 0.174 |
| -51 | 0.826 | 0.150 | 0.014 | 0.005 | 0.005 | 0.174 |
| -50 | 0.826 | 0.150 | 0.014 | 0.005 | 0.005 | 0.174 |
| -49 | 0.826 | 0.150 | 0.014 | 0.005 | 0.005 | 0.174 |
| -48 | 0.826 | 0.150 | 0.014 | 0.005 | 0.005 | 0.174 |
| -47 | 0.825 | 0.150 | 0.014 | 0.005 | 0.005 | 0.175 |
| -46 | 0.825 | 0.150 | 0.014 | 0.005 | 0.005 | 0.175 |
| -45 | 0.825 | 0.150 | 0.014 | 0.005 | 0.005 | 0.175 |
| -44 | 0.825 | 0.150 | 0.014 | 0.005 | 0.005 | 0.175 |
| -43 | 0.825 | 0.151 | 0.014 | 0.005 | 0.005 | 0.175 |
| -42 | 0.824 | 0.151 | 0.014 | 0.005 | 0.006 | 0.176 |
| -41 | 0.824 | 0.151 | 0.014 | 0.005 | 0.006 | 0.176 |
| -40 | 0.824 | 0.151 | 0.014 | 0.005 | 0.006 | 0.176 |
| -39 | 0.824 | 0.151 | 0.014 | 0.005 | 0.006 | 0.176 |
| -38 | 0.824 | 0.151 | 0.014 | 0.005 | 0.006 | 0.176 |
| -37 | 0.825 | 0.151 | 0.014 | 0.005 | 0.005 | 0.175 |
| -36 | 0.825 | 0.151 | 0.014 | 0.005 | 0.005 | 0.175 |
| -35 | 0.825 | 0.151 | 0.014 | 0.005 | 0.005 | 0.175 |
| -34 | 0.824 | 0.151 | 0.014 | 0.005 | 0.006 | 0.176 |
| -33 | 0.823 | 0.152 | 0.014 | 0.005 | 0.006 | 0.177 |
| -32 | 0.822 | 0.153 | 0.014 | 0.005 | 0.006 | 0.178 |
| -31 | 0.821 | 0.154 | 0.014 | 0.005 | 0.006 | 0.179 |
| -30 | 0.819 | 0.155 | 0.015 | 0.005 | 0.006 | 0.181 |
| -29 | 0.819 | 0.156 | 0.015 | 0.005 | 0.006 | 0.181 |
| -28 | 0.818 | 0.156 | 0.015 | 0.005 | 0.006 | 0.182 |
| -27 | 0.818 | 0.156 | 0.015 | 0.005 | 0.006 | 0.182 |
| -26 | 0.818 | 0.156 | 0.015 | 0.005 | 0.006 | 0.182 |
| -25 | 0.818 | 0.156 | 0.015 | 0.005 | 0.006 | 0.182 |
| -24 | 0.818 | 0.156 | 0.015 | 0.005 | 0.006 | 0.182 |
| -23 | 0.818 | 0.156 | 0.015 | 0.005 | 0.006 | 0.182 |
| -22 | 0.818 | 0.156 | 0.015 | 0.005 | 0.006 | 0.182 |
| -21 | 0.818 | 0.156 | 0.015 | 0.005 | 0.006 | 0.182 |
| -20 | 0.817 | 0.157 | 0.015 | 0.005 | 0.006 | 0.183 |
| -19 | 0.817 | 0.157 | 0.015 | 0.005 | 0.006 | 0.183 |
| -18 | 0.816 | 0.158 | 0.015 | 0.005 | 0.006 | 0.184 |
| -17 | 0.815 | 0.159 | 0.015 | 0.005 | 0.006 | 0.185 |
| -16 | 0.814 | 0.160 | 0.015 | 0.006 | 0.006 | 0.186 |
| -15 | 0.812 | 0.161 | 0.015 | 0.006 | 0.006 | 0.188 |
| -14 | 0.811 | 0.162 | 0.015 | 0.006 | 0.006 | 0.189 |
| -13 | 0.810 | 0.163 | 0.016 | 0.006 | 0.006 | 0.190 |
| -12 | 0.809 | 0.164 | 0.016 | 0.006 | 0.006 | 0.191 |
| -11 | 0.809 | 0.164 | 0.016 | 0.006 | 0.006 | 0.191 |
| -10 | 0.809 | 0.164 | 0.016 | 0.006 | 0.006 | 0.191 |
| -9 | 0.810 | 0.163 | 0.016 | 0.006 | 0.006 | 0.190 |
| -8 | 0.811 | 0.162 | 0.015 | 0.006 | 0.006 | 0.189 |
| -7 | 0.812 | 0.161 | 0.015 | 0.006 | 0.006 | 0.188 |
| -6 | 0.812 | 0.161 | 0.015 | 0.006 | 0.006 | 0.188 |
| -5 | 0.811 | 0.162 | 0.015 | 0.006 | 0.006 | 0.189 |
| -4 | 0.809 | 0.164 | 0.016 | 0.006 | 0.006 | 0.191 |
| -3 | 0.805 | 0.167 | 0.016 | 0.006 | 0.006 | 0.195 |
| -2 | 0.798 | 0.173 | 0.017 | 0.006 | 0.007 | 0.202 |
| -1 | 0.788 | 0.181 | 0.018 | 0.006 | 0.007 | 0.212 |
| 0 | 0.778 | 0.189 | 0.019 | 0.007 | 0.007 | 0.222 |
| 1 | 0.768 | 0.197 | 0.020 | 0.007 | 0.008 | 0.232 |
| 2 | 0.761 | 0.203 | 0.020 | 0.008 | 0.008 | 0.239 |
| 3 | 0.758 | 0.206 | 0.021 | 0.008 | 0.008 | 0.242 |
| 4 | 0.759 | 0.205 | 0.021 | 0.008 | 0.008 | 0.241 |
| 5 | 0.763 | 0.201 | 0.020 | 0.007 | 0.008 | 0.237 |
| 6 | 0.769 | 0.196 | 0.020 | 0.007 | 0.008 | 0.231 |
| 7 | 0.776 | 0.190 | 0.019 | 0.007 | 0.007 | 0.224 |
| 8 | 0.783 | 0.185 | 0.018 | 0.007 | 0.007 | 0.217 |
| 9 | 0.789 | 0.180 | 0.018 | 0.006 | 0.007 | 0.211 |
| 10 | 0.793 | 0.177 | 0.017 | 0.006 | 0.007 | 0.207 |
| 11 | 0.795 | 0.175 | 0.017 | 0.006 | 0.007 | 0.205 |
| 12 | 0.796 | 0.174 | 0.017 | 0.006 | 0.007 | 0.204 |
| 13 | 0.796 | 0.174 | 0.017 | 0.006 | 0.007 | 0.204 |
| 14 | 0.796 | 0.174 | 0.017 | 0.006 | 0.007 | 0.204 |
| 15 | 0.796 | 0.175 | 0.017 | 0.006 | 0.007 | 0.204 |
| 16 | 0.796 | 0.174 | 0.017 | 0.006 | 0.007 | 0.204 |
| 17 | 0.797 | 0.174 | 0.017 | 0.006 | 0.007 | 0.203 |
| 18 | 0.797 | 0.173 | 0.017 | 0.006 | 0.007 | 0.203 |
| 19 | 0.798 | 0.173 | 0.017 | 0.006 | 0.007 | 0.202 |
| 20 | 0.798 | 0.173 | 0.017 | 0.006 | 0.007 | 0.202 |
| 21 | 0.797 | 0.173 | 0.017 | 0.006 | 0.007 | 0.203 |
| 22 | 0.797 | 0.173 | 0.017 | 0.006 | 0.007 | 0.203 |
| 23 | 0.797 | 0.174 | 0.017 | 0.006 | 0.007 | 0.203 |
| 24 | 0.796 | 0.174 | 0.017 | 0.006 | 0.007 | 0.204 |
| 25 | 0.796 | 0.175 | 0.017 | 0.006 | 0.007 | 0.204 |
| 26 | 0.795 | 0.175 | 0.017 | 0.006 | 0.007 | 0.205 |
| 27 | 0.794 | 0.176 | 0.017 | 0.006 | 0.007 | 0.206 |
| 28 | 0.792 | 0.178 | 0.017 | 0.006 | 0.007 | 0.208 |
| 29 | 0.791 | 0.179 | 0.017 | 0.006 | 0.007 | 0.209 |
| 30 | 0.789 | 0.180 | 0.018 | 0.006 | 0.007 | 0.211 |
| 31 | 0.789 | 0.180 | 0.018 | 0.006 | 0.007 | 0.211 |
| 32 | 0.789 | 0.181 | 0.018 | 0.006 | 0.007 | 0.211 |
| 33 | 0.789 | 0.180 | 0.018 | 0.006 | 0.007 | 0.211 |
| 34 | 0.790 | 0.179 | 0.018 | 0.006 | 0.007 | 0.210 |
| 35 | 0.790 | 0.179 | 0.017 | 0.006 | 0.007 | 0.210 |
| 36 | 0.791 | 0.179 | 0.017 | 0.006 | 0.007 | 0.209 |
| 37 | 0.791 | 0.179 | 0.017 | 0.006 | 0.007 | 0.209 |
| 38 | 0.790 | 0.179 | 0.017 | 0.006 | 0.007 | 0.210 |
| 39 | 0.789 | 0.180 | 0.018 | 0.006 | 0.007 | 0.211 |
| 40 | 0.789 | 0.180 | 0.018 | 0.006 | 0.007 | 0.211 |
| 41 | 0.788 | 0.181 | 0.018 | 0.006 | 0.007 | 0.212 |
| 42 | 0.787 | 0.182 | 0.018 | 0.006 | 0.007 | 0.213 |
| 43 | 0.787 | 0.182 | 0.018 | 0.007 | 0.007 | 0.213 |
| 44 | 0.787 | 0.182 | 0.018 | 0.007 | 0.007 | 0.213 |
| 45 | 0.787 | 0.182 | 0.018 | 0.007 | 0.007 | 0.213 |
| 46 | 0.788 | 0.181 | 0.018 | 0.006 | 0.007 | 0.212 |
| 47 | 0.788 | 0.181 | 0.018 | 0.006 | 0.007 | 0.212 |
| 48 | 0.789 | 0.180 | 0.018 | 0.006 | 0.007 | 0.211 |
| 49 | 0.789 | 0.180 | 0.018 | 0.006 | 0.007 | 0.211 |
| 50 | 0.789 | 0.180 | 0.018 | 0.006 | 0.007 | 0.211 |
| 51 | 0.789 | 0.180 | 0.018 | 0.006 | 0.007 | 0.211 |
| 52 | 0.789 | 0.180 | 0.018 | 0.006 | 0.007 | 0.211 |
| 53 | 0.790 | 0.179 | 0.018 | 0.006 | 0.007 | 0.210 |
| 54 | 0.790 | 0.179 | 0.017 | 0.006 | 0.007 | 0.210 |
| 55 | 0.790 | 0.180 | 0.018 | 0.006 | 0.007 | 0.210 |
| 56 | 0.789 | 0.180 | 0.018 | 0.006 | 0.007 | 0.211 |
| 57 | 0.787 | 0.182 | 0.018 | 0.006 | 0.007 | 0.213 |
| 58 | 0.786 | 0.183 | 0.018 | 0.007 | 0.007 | 0.214 |
| 59 | 0.785 | 0.184 | 0.018 | 0.007 | 0.007 | 0.215 |
| 60 | 0.784 | 0.184 | 0.018 | 0.007 | 0.007 | 0.216 |
| 61 | 0.785 | 0.184 | 0.018 | 0.007 | 0.007 | 0.215 |
| 62 | 0.786 | 0.183 | 0.018 | 0.007 | 0.007 | 0.214 |
| 63 | 0.786 | 0.182 | 0.018 | 0.007 | 0.007 | 0.214 |
| 64 | 0.787 | 0.182 | 0.018 | 0.007 | 0.007 | 0.213 |
| 65 | 0.786 | 0.182 | 0.018 | 0.007 | 0.007 | 0.214 |
| 66 | 0.786 | 0.183 | 0.018 | 0.007 | 0.007 | 0.214 |
| 67 | 0.785 | 0.184 | 0.018 | 0.007 | 0.007 | 0.215 |
| 68 | 0.784 | 0.184 | 0.018 | 0.007 | 0.007 | 0.216 |
| 69 | 0.783 | 0.185 | 0.018 | 0.007 | 0.007 | 0.217 |
| 70 | 0.783 | 0.185 | 0.018 | 0.007 | 0.007 | 0.217 |
| 71 | 0.783 | 0.185 | 0.018 | 0.007 | 0.007 | 0.217 |
| 72 | 0.784 | 0.185 | 0.018 | 0.007 | 0.007 | 0.216 |
| 73 | 0.784 | 0.184 | 0.018 | 0.007 | 0.007 | 0.216 |
| 74 | 0.784 | 0.184 | 0.018 | 0.007 | 0.007 | 0.216 |
| 75 | 0.784 | 0.184 | 0.018 | 0.007 | 0.007 | 0.216 |
| 76 | 0.783 | 0.185 | 0.018 | 0.007 | 0.007 | 0.217 |
| 77 | 0.782 | 0.186 | 0.018 | 0.007 | 0.007 | 0.218 |
| 78 | 0.780 | 0.187 | 0.018 | 0.007 | 0.007 | 0.220 |
| 79 | 0.779 | 0.188 | 0.019 | 0.007 | 0.007 | 0.221 |
| 80 | 0.778 | 0.189 | 0.019 | 0.007 | 0.007 | 0.222 |
| 81 | 0.778 | 0.189 | 0.019 | 0.007 | 0.007 | 0.222 |
| 82 | 0.778 | 0.189 | 0.019 | 0.007 | 0.007 | 0.222 |
| 83 | 0.777 | 0.190 | 0.019 | 0.007 | 0.007 | 0.223 |
| 84 | 0.777 | 0.190 | 0.019 | 0.007 | 0.007 | 0.223 |
| 85 | 0.776 | 0.191 | 0.019 | 0.007 | 0.007 | 0.224 |
| 86 | 0.776 | 0.191 | 0.019 | 0.007 | 0.007 | 0.224 |
| 87 | 0.776 | 0.191 | 0.019 | 0.007 | 0.007 | 0.224 |
| 88 | 0.776 | 0.191 | 0.019 | 0.007 | 0.007 | 0.224 |
| 89 | 0.777 | 0.190 | 0.019 | 0.007 | 0.007 | 0.223 |
| 90 | 0.777 | 0.190 | 0.019 | 0.007 | 0.007 | 0.223 |
| 91 | 0.778 | 0.189 | 0.019 | 0.007 | 0.007 | 0.222 |
| 92 | 0.778 | 0.189 | 0.019 | 0.007 | 0.007 | 0.222 |
| 93 | 0.778 | 0.189 | 0.019 | 0.007 | 0.007 | 0.222 |
| 94 | 0.778 | 0.189 | 0.019 | 0.007 | 0.007 | 0.222 |
| 95 | 0.778 | 0.189 | 0.019 | 0.007 | 0.007 | 0.222 |
| 96 | 0.778 | 0.189 | 0.019 | 0.007 | 0.007 | 0.222 |
| 97 | 0.778 | 0.189 | 0.019 | 0.007 | 0.007 | 0.222 |
| 98 | 0.778 | 0.189 | 0.019 | 0.007 | 0.007 | 0.222 |
| 99 | 0.778 | 0.189 | 0.019 | 0.007 | 0.007 | 0.222 |
| 100 | 0.778 | 0.189 | 0.019 | 0.007 | 0.007 | 0.222 |
| 101 | 0.777 | 0.190 | 0.019 | 0.007 | 0.007 | 0.223 |
| 102 | 0.777 | 0.190 | 0.019 | 0.007 | 0.007 | 0.223 |
| 103 | 0.776 | 0.191 | 0.019 | 0.007 | 0.007 | 0.224 |
| 104 | 0.775 | 0.191 | 0.019 | 0.007 | 0.007 | 0.225 |
| 105 | 0.775 | 0.192 | 0.019 | 0.007 | 0.007 | 0.225 |
| 106 | 0.774 | 0.192 | 0.019 | 0.007 | 0.008 | 0.226 |
| 107 | 0.774 | 0.192 | 0.019 | 0.007 | 0.008 | 0.226 |
| 108 | 0.774 | 0.192 | 0.019 | 0.007 | 0.008 | 0.226 |
| 109 | 0.774 | 0.192 | 0.019 | 0.007 | 0.008 | 0.226 |
| 110 | 0.774 | 0.192 | 0.019 | 0.007 | 0.008 | 0.226 |
| 111 | 0.774 | 0.192 | 0.019 | 0.007 | 0.008 | 0.226 |
| 112 | 0.774 | 0.192 | 0.019 | 0.007 | 0.008 | 0.226 |
| 113 | 0.774 | 0.192 | 0.019 | 0.007 | 0.008 | 0.226 |
| 114 | 0.773 | 0.193 | 0.019 | 0.007 | 0.008 | 0.227 |
| 115 | 0.772 | 0.194 | 0.019 | 0.007 | 0.008 | 0.228 |
| 116 | 0.771 | 0.195 | 0.019 | 0.007 | 0.008 | 0.229 |
| 117 | 0.770 | 0.196 | 0.020 | 0.007 | 0.008 | 0.230 |
| 118 | 0.770 | 0.196 | 0.020 | 0.007 | 0.008 | 0.230 |
| 119 | 0.771 | 0.195 | 0.019 | 0.007 | 0.008 | 0.229 |
| 120 | 0.772 | 0.194 | 0.019 | 0.007 | 0.008 | 0.228 |

Supplementary table 1: Table of modelled categorical responses for physical contacts with 18 to 69 year olds and its translation to a probability of having had some contacts, i.e. summing response categories 2 to 5.

**Testing behavioural differences between ChAdOx1 versus the BNT162b2 vaccines**


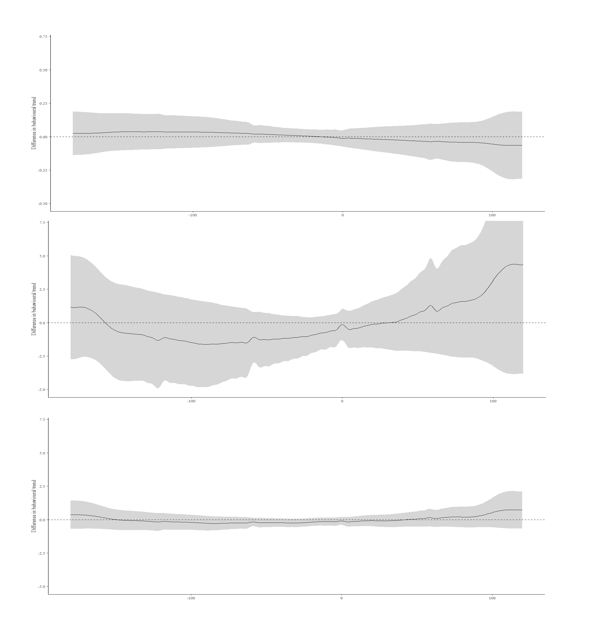


Supplementary figure 9: Differences in smooths between ChAdOx1 versus the BNT162b2 vaccines for adults aged 18-64 years. Top panel: others in own home in the past 7 days. Middle panel: physical contacts with 18 to 69 year-olds in the past 7 days. Bottom panel: Socially-distanced contacts with 18 to 69 year-olds in the past 7 days. No evidence to suggest behavioural trends diverge between individuals that received either ChAdOx1 versus the BNT162b2 vaccines.

**Behavioural response to second vaccination**


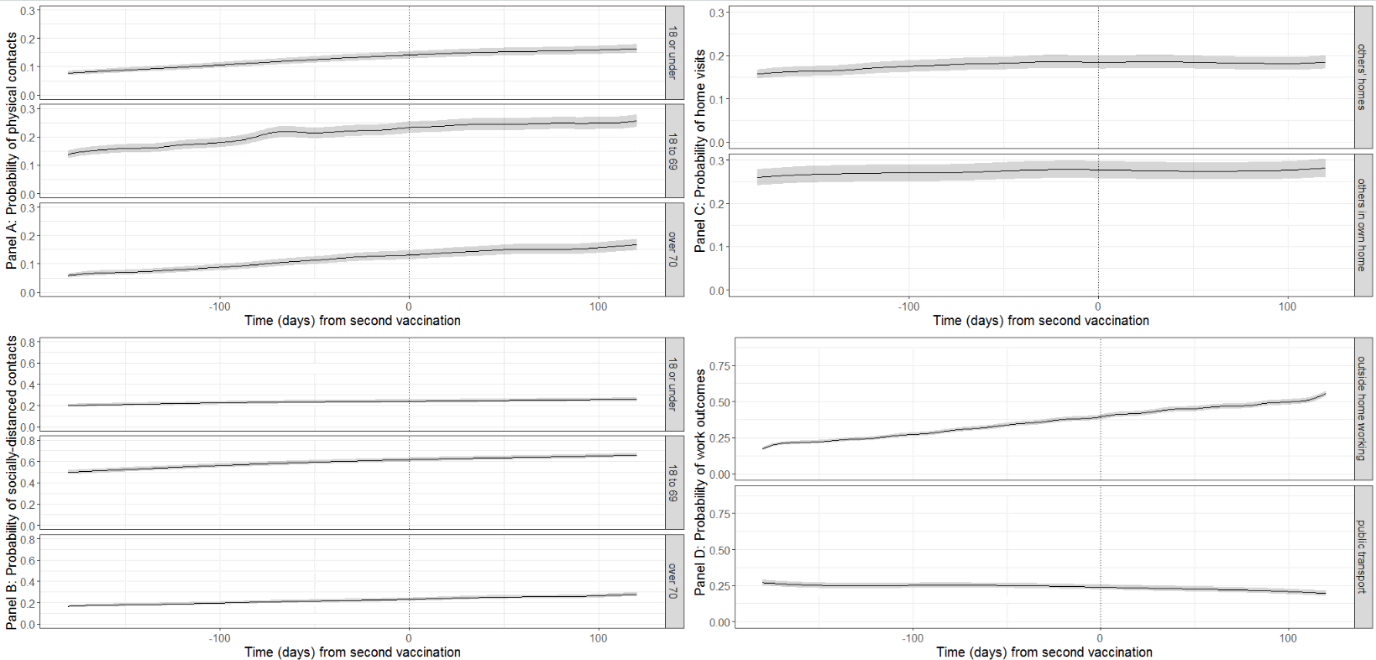


Supplementary figure 10: Probabilities of behavioural outcomes for individuals aged 18-64y by time since second vaccination. Top left (panel A): past 7-day reported physical, outside of household contacts; bottom left (Panel B): past 7-day reported socially-distanced, outside of household contacts; top right (Panel C): past 7-day reported home visits; bottom right (Panel D): past 7-day reported work outcomes for those that are working or in education. Dotted line shows day of own first vaccination. “18 or under”, “18 to 69” and “over 70” denote the ages of the people that individuals in the sample had contact with.


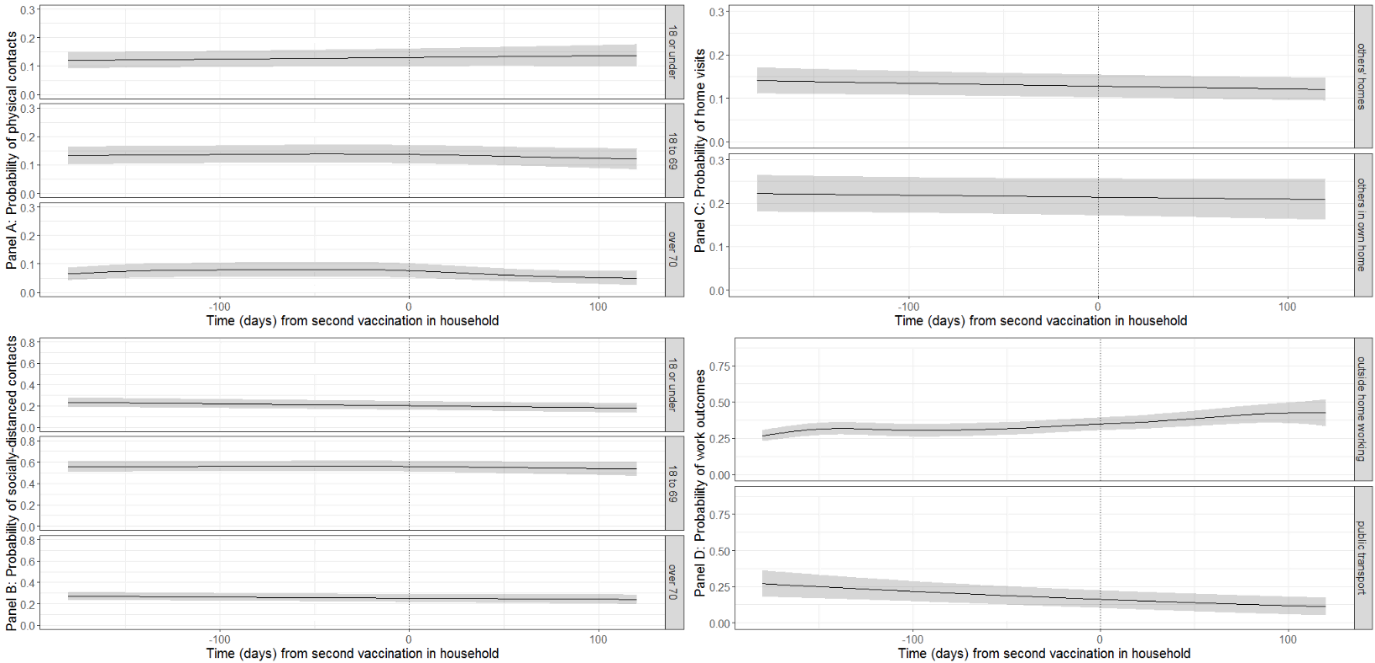


Supplementary figure 11: Probabilities of behavioural outcomes for unvaccinated individuals aged 18-64y by time to the first day that all vulnerable household members are vaccinated with at least 2 vaccinations. Vulnerable household members are defined as defined as aged 65 and over or had a long-term health condition, in keeping with vaccine allocation groups of the UK government. Top left (panel A): past 7-day reported physical, outside of household contacts; bottom left (Panel B): past 7-day reported socially-distanced, outside of household contacts; top right (Panel C): past 7-day reported home visits; bottom right (Panel D): past 7-day reported work outcomes for those that are working or in education. Dotted line shows day of own first vaccination. “18 or under”, “18 to 69” and “over 70” denote the ages of the people that individuals in the sample had contact with.


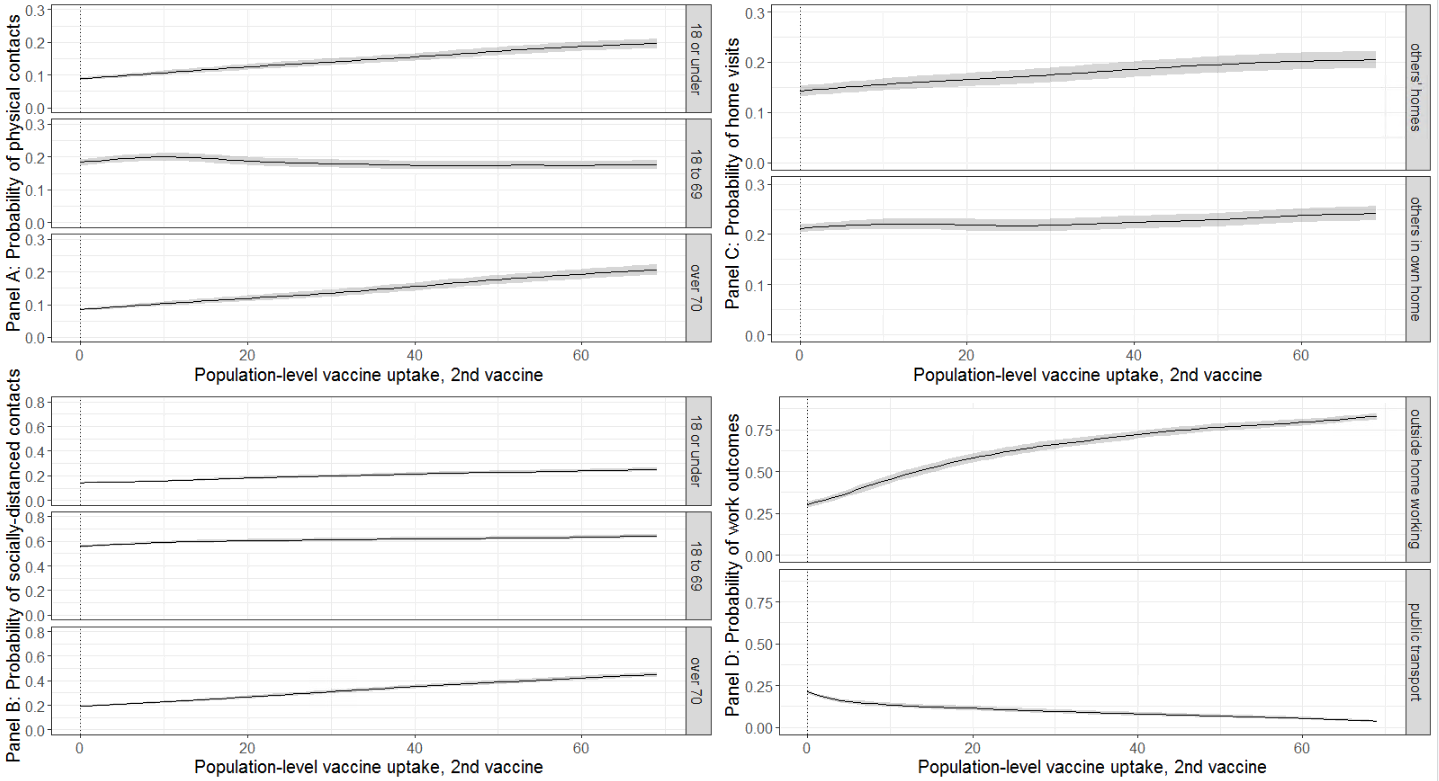


Supplementary figure 12: Probabilities of behavioural outcomes by population level vaccination %, second vaccination. Top left (panel A): past 7-day reported physical, outside of household contacts; bottom left (Panel B): past 7-day reported socially-distanced, outside of household contacts; top right (Panel C): past 7-day reported home visits; bottom right (Panel D): past 7-day reported work outcomes for those that are working or in education. “18 or under”, “18 to 69” and “over 70” denote the ages of the people that individuals in the sample had contact with.

**Analysis of population level vaccination restricted to the sample used for response to own vaccination**

Supplementary figure 13 shows the variation in 10 behavioural outcomes as a function of the rate of first vaccination in the population, using the sample of vaccinated individuals as per individual vaccination.

The probability of outside of the household - physical and socially-distanced - contacts increased as population level vaccination increased (Supplementary figure 13 panels A and B). In contrast to the full sample, an initial peak in the probability of contacts in the first 25% of population vaccination is not observed. Probabilities of home visits appeared to be stable as population vaccination rates increased (Supplementary figure 13 panel C). Probabilities of both working at home and taking public transport decreased as population vaccination rates increased (Supplementary figure 13 panel D).


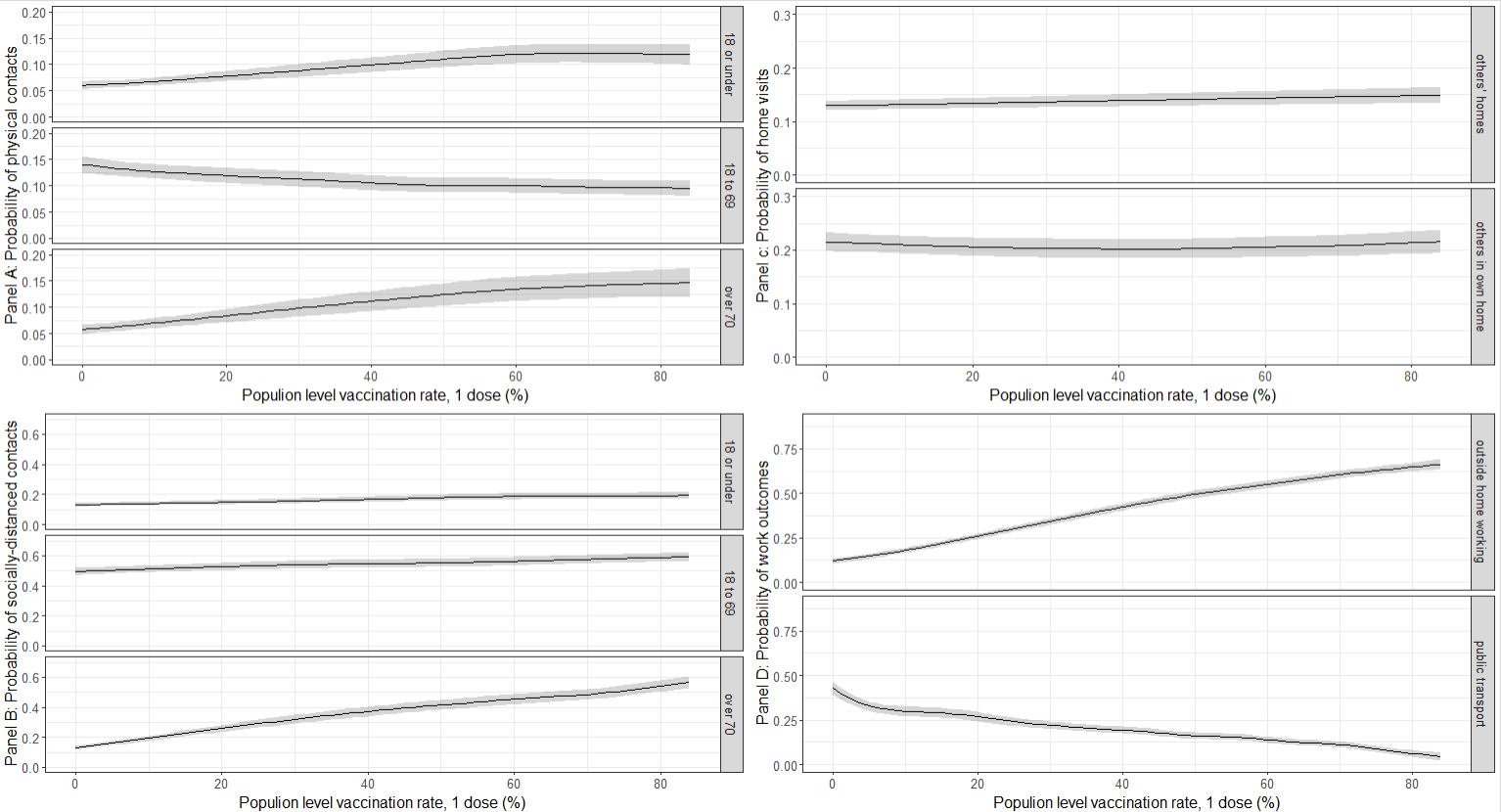


Supplementary figure 13: Probabilities of behavioural outcomes by population level vaccination %, first dose. Top left (panel A): past 7-day reported physical, outside of household contacts; bottom left (Panel B): past 7-day reported socially-distanced, outside of household contacts; top right (Panel C): past 7-day reported home visits; bottom right (Panel D): past 7-day reported work outcomes for those that are working or in education. “18 or under”, “18 to 69” and “over 70” denote the ages of the people that individuals in the sample had contact with.

**Counts of responses to outcomes, own vaccination of 18 to 64 year-olds**

| Outcome | Behaviour captured by binary variable | Counts | | | | | | | | | | | | |
| --- | --- | --- | --- | --- | --- | --- | --- | --- | --- | --- | --- | --- | --- | --- |
|  |  | 0 | 1 to 5 | 6 to 10 | 11 to 20 | 21 or more |  |  |  | any | n | missing | denominator | % any |
|  |  |  |  |  |  |  |  |  |  |  |  |  |  |  |
| Physical contacts with under 18s | Any  past 7-day contacts outside the household | 1418281 | 307686 | 22615 | 13476 | 34547 |  |  |  | 421630 | 1839911 | 43306 | 1796605 | 23.5% |
| Physical contacts with 18 to 69 years | Any past 7-day contacts outside the household | 1153840 | 516900 | 70029 | 27323 | 30369 |  |  |  | 686071 | 1839911 | 41423 | 1798488 | 38.1% |
| Physical contacts with over 70s | Any past 7-day contacts outside the household | 1524370 | 251502 | 8050 | 3848 | 7045 |  |  |  | 315541 | 1839911 | 45096 | 1794815 | 17.6% |
| Socially-distanced contacts with under 18s | Any past 7-day contacts outside the household | 1226960 | 324924 | 69179 | 39969 | 132484 |  |  |  | 612951 | 1839911 | 46395 | 1793516 | 34.2% |
| Socially-distanced contacts with 18 to 69 years | Any past 7-day contacts outside the household | 483189 | 650509 | 257043 | 158173 | 249222 |  |  |  | 1356722 | 1839911 | 41775 | 1798136 | 75.5% |
| Socially-distanced contacts with over 70s | Any past 7-day contacts outside the household | 1226525 | 437158 | 48386 | 26341 | 54933 |  |  |  | 613386 | 1839911 | 46568 | 1793343 | 34.2% |
|  |  |  |  |  |  |  |  |  |  |  |  |  |  |  |
|  |  | 0 | 1 | 2 | 3 | 4 | 5 | 6 | 7 or more |  |  |  |  |  |
| Visits to others' homes | Any past 7-day home visits | 1226558 | 276882 | 90513 | 34616 | 21964 | 15607 | 13091 | 4392 | 613353 | 1839911 | 156288 | 1683623 | 36.4% |
| Others' visits to own home | Any past 7-day home visits | 1153551 | 313759 | 111641 | 44129 | 18670 | 20109 | 15295 | 4774 | 686360 | 1839911 | 157983 | 1681928 | 40.8% |
|  |  |  |  |  |  |  |  |  |  |  |  |  |  |  |
|  |  | Working from home | | Mix of home outside | | Working outside home | |  |  |  |  |  |  |  |
| Working/studying outside home | Any past 7-day working outside home | 455669 | | 129046 | | 392625 | |  |  | 521671 | 1009555 | 32215 | 977340 | 53.4% |
|  |  |  |  |  |  |  |  |  |  |  |  |  |  |  |
|  |  | Private transport | | Public transport | |  |  |  |  |  |  |  |  |  |
| Taking public transport to work/place of education | Any past 7-day public transport | 725771 | | 76021 | |  |  |  |  | 76021 | 1009555 | 207762 | 801793 | 9.5% |
|  |  |  |  |  |  |  |  |  |  |  |  |  |  |  |

Supplementary table 2: Counts of self-reported behaviours across all outcomes. The numerator is the number of observations for which “any” was reported and the denominator is the total number of observations in that analysis. Denominators for work variables restricted to those reporting working or in education. Zero – counts of “none”, any – counts of “any”, n – sample size in analysis, missing – missing outcome (not reported), denominator – sample size less missing observations, % any – percentage of observations reporting “any”.

**Counts of responses to outcomes, household vaccination of 18 to 64 year-olds**

| Outcome | Behaviour captured by binary variable | Counts | | | | | | | | | | | | |
| --- | --- | --- | --- | --- | --- | --- | --- | --- | --- | --- | --- | --- | --- | --- |
|  |  | zero | 1 to 5 | 6 to 10 | 11 to 20 | 21 or more |  |  |  | any | n | missing | denominator | % any |
|  |  |  |  |  |  |  |  |  |  |  |  |  |  |  |
| Physical contacts with under 18s | Any  past 7-day contacts outside the household | 42198 | 8199 | 429 | 294 | 826 |  |  |  | 11648 | 53846 | 1900 | 51946 | 22.4% |
| Physical contacts with 18 to 69 years | Any past 7-day contacts outside the household | 36745 | 12833 | 1238 | 547 | 628 |  |  |  | 17101 | 53846 | 1805 | 52041 | 32.9% |
| Physical contacts with over 70s | Any past 7-day contacts outside the household | 46099 | 5436 | 144 | 67 | 143 |  |  |  | 7747 | 53846 | 1957 | 51889 | 14.9% |
| Socially-distanced contacts with under 18s | Any past 7-day contacts outside the household | 38121 | 8256 | 1452 | 890 | 3116 |  |  |  | 15725 | 53846 | 2011 | 51835 | 30.3% |
| Socially-distanced contacts with 18 to 69 years | Any past 7-day contacts outside the household | 16334 | 18996 | 6358 | 3952 | 6419 |  |  |  | 37512 | 53846 | 1787 | 52059 | 72.1% |
| Socially-distanced contacts with over 70s | Any past 7-day contacts outside the household | 36947 | 11371 | 1279 | 666 | 1541 |  |  |  | 16899 | 53846 | 2042 | 51804 | 32.6% |
|  |  |  |  |  |  |  |  |  |  |  |  |  |  |  |
|  |  | 0 | 1 | 2 | 3 | 4 | 5 | 6 | 7 or more |  |  |  |  |  |
| Visits to others' homes | Any past 7-day home visits | 37235 | 5919 | 1660 | 623 | 477 | 292 | 294 | 85 | 16611 | 53846 | 7261 | 46585 | 35.7% |
| Others' visits to own home | Any past 7-day home visits | 34912 | 7238 | 2338 | 822 | 412 | 398 | 300 | 97 | 18934 | 53846 | 7329 | 46517 | 40.7% |
|  |  |  |  |  |  |  |  |  |  |  |  |  |  |  |
|  |  | Working from home | | Mix of home outside | | Working outside home | |  |  |  |  |  |  |  |
| Working/studying outside home | Any past 7-day working outside home | 29721 | | 5940 | | 28732 | |  |  | 34672 | 67531 | 3138 | 64393 | 53.8% |
|  |  |  |  |  |  |  |  |  |  |  |  |  |  |  |
|  |  | Private transport | | Public transport | |  |  |  |  |  |  |  |  |  |
| Taking public transport to work/place of education | Any past 7-day public transport | 37973 | | 3633 | |  |  |  |  | 3633 | 67531 | 25925 | 41606 | 8.7% |
|  |  |  |  |  |  |  |  |  |  |  |  |  |  |  |

Supplementary table 3: Counts of self-reported behaviours across all outcomes. The numerator is the number of observations for which “any” was reported and the denominator is the total number of observations in that analysis. Denominators for work variables restricted to those reporting working or in education. Zero – counts of “none”, any – counts of “any”, n – sample size in analysis, missing – missing outcome (not reported), denominator – sample size less missing observations, % any – percentage of observations reporting “any”.

**Counts of responses to outcomes, population level vaccination of all ages**

| Outcome | Behaviour captured by binary variable | Counts | | | | | | | | | | | | |
| --- | --- | --- | --- | --- | --- | --- | --- | --- | --- | --- | --- | --- | --- | --- |
|  |  | 0 | 1 to 5 | 6 to 10 | 11 to 20 | 21 or more |  |  |  | any | n | missing | denominator | % any |
|  |  |  |  |  |  |  |  |  |  |  |  |  |  |  |
| Physical contacts with under 18s | Any  past 7-day contacts outside the household | 3200446 | 830713 | 105033 | 76693 | 196582 |  |  |  | 1308309 | 4508755 | 99288 | 4409467 | 29.7% |
| Physical contacts with 18 to 69 years | Any past 7-day contacts outside the household | 2755714 | 1305710 | 188480 | 72520 | 90354 |  |  |  | 1753041 | 4508755 | 95977 | 4412778 | 39.7% |
| Physical contacts with over 70s | Any past 7-day contacts outside the household | 3700848 | 614663 | 33213 | 20122 | 36040 |  |  |  | 807907 | 4508755 | 103869 | 4404886 | 18.3% |
| Socially-distanced contacts with under 18s | Any past 7-day contacts outside the household | 2913325 | 784980 | 156076 | 102837 | 445076 |  |  |  | 1595430 | 4508755 | 106461 | 4402294 | 36.2% |
| Socially-distanced contacts with 18 to 69 years | Any past 7-day contacts outside the household | 1324056 | 1660861 | 596757 | 336088 | 493633 |  |  |  | 3184699 | 4508755 | 97360 | 4411395 | 72.2% |
| Socially-distanced contacts with over 70s | Any past 7-day contacts outside the household | 2994133 | 1058919 | 139069 | 74368 | 134860 |  |  |  | 1514622 | 4508755 | 107406 | 4401349 | 34.4% |
|  |  |  |  |  |  |  |  |  |  |  |  |  |  |  |
|  |  | 0 | 1 | 2 | 3 | 4 | 5 | 6 | 7 or more |  |  |  |  |  |
| Visits to others' homes | Any past 7-day home visits | 3057637 | 665548 | 218948 | 84669 | 50519 | 38434 | 31273 | 10540 | 1451118 | 4508755 | 351187 | 4157568 | 34.9% |
| Others' visits to own home | Any past 7-day home visits | 2826878 | 783615 | 281142 | 112403 | 48333 | 51927 | 36940 | 12526 | 1681877 | 4508755 | 354991 | 4153764 | 40.5% |
|  |  |  |  |  |  |  |  |  |  |  |  |  |  |  |
|  |  | Working from home | | Mix of home outside | | Working outside home | |  |  |  |  |  |  |  |
| Working/studying outside home | Any past 7-day working outside home | 885522 | | 252424 | | 910658 | |  |  | 1163082 | 2705321 | 656717 | 2048604 | 56.8% |
|  |  |  |  |  |  |  |  |  |  |  |  |  |  |  |
|  |  | Private transport | | Public transport | |  |  |  |  |  |  |  |  |  |
| Taking public transport to work/place of education | Any past 7-day public transport | 1574879 | | 195547 | |  |  |  |  | 195547 | 2705321 | 934895 | 1770426 | 11.0% |
|  |  |  |  |  |  |  |  |  |  |  |  |  |  |  |

Supplementary table 4: Counts of self-reported behaviours across all outcomes. The numerator is the number of observations for which “any” was reported and the denominator is the total number of observations in that analysis. Denominators for work variables restricted to those reporting working or in education. Zero – counts of “none”, any – counts of “any”, n – sample size in analysis, missing – missing outcome (not reported), denominator – sample size less missing observations, % any – percentage of observations reporting “any”

**Analysis of time and national rollout of vaccination**

|  |  | Outcome | | |
| --- | --- | --- | --- | --- |
|  |  | (a) physical contacts18 to 64 | (b) social contacts 18 to 64 | (c) times in others' homes |
| 1 | vaccine and time | 7611764 | 12057297 | 7089612 |
| 2 | vaccine only | 7668678 | 12087081 | 7142979 |
| 3 | time only | 7617305 | 12087775 | 7092030 |

Supplementary table 5: Table of AICs for 9 models. Three outcomes are physical contacts18 to 64 (a), socially distanced contacts 18 to 64 (b), and the number of visits to others’ homes (c). For each outcome, 3 specifications are used: proportion of the population that had been vaccinated and calendar time (1); proportion of the population that had been vaccinated only (2); and calendar time only (3).


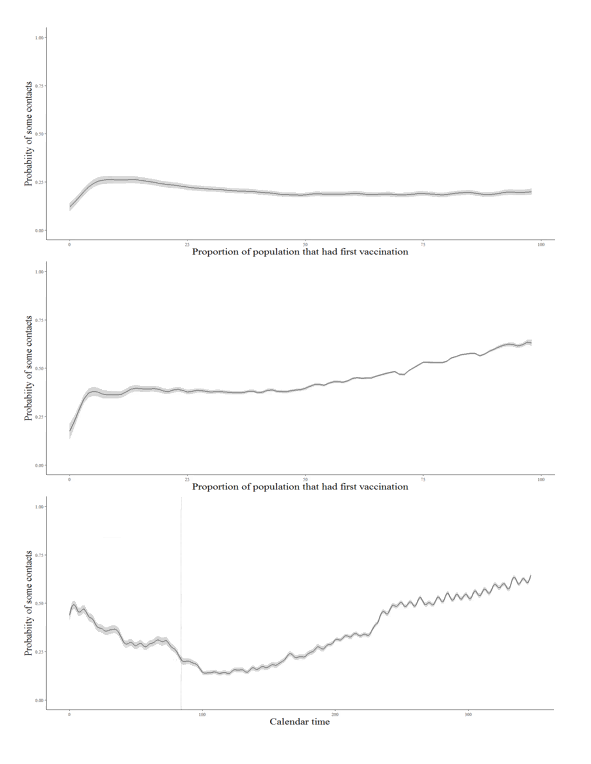


Supplementary figure 14: Probabilities of having any physical contacts with individuals aged 18-64 for three model specifications. Top panel - proportion of the population that had been vaccinated and calendar time (model 1a); NB – x-axis is proportion of the population that had their first vaccine. Middle panel - proportion of the population that had been vaccinated only (model 2a) ; NB – x-axis is proportion of the population that had their first vaccine. Lower panel - calendar time only (model 3a) ; NB – x-axis is calendar time, where day 68 (dotted vertical line) was the beginning of the vaccine rollout.


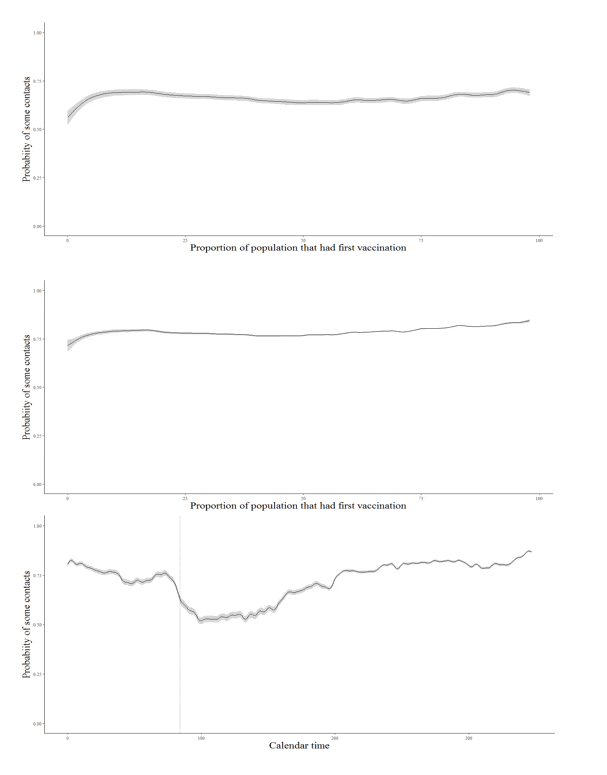


Supplementary figure 15: Probabilities of having any socially-distanced contacts with individuals aged 18-64 for three model specifications. Top panel - proportion of the population that had been vaccinated and calendar time (model 1b); NB – x-axis is proportion of the population that had their first vaccine. Middle panel - proportion of the population that had been vaccinated only (model 2b); NB – x-axis is proportion of the population that had their first vaccine. Lower panel - calendar time only (model 3b); NB – x-axis is calendar time, where day 68 (dotted vertical line) was the beginning of the vaccine rollout.


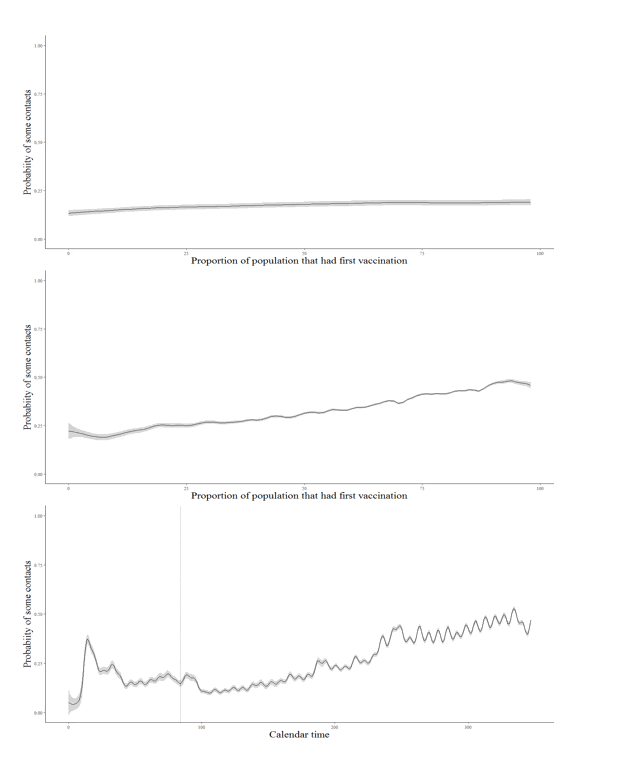


Supplementary figure 16: Number of visits to others’ homes for three model specifications. Top panel - proportion of the population that had been vaccinated and calendar time (model 1c); NB – x-axis is proportion of the population that had their first vaccine. Middle panel - proportion of the population that had been vaccinated only (model 2c); NB – x-axis is proportion of the population that had their first vaccine. Lower panel - calendar time only (model 3c); NB – x-axis is calendar time, where day 68 (dotted vertical line) was the beginning of the vaccine rollout.

**Analysis of weekly and fortnightly time alongside national rollout of vaccination**


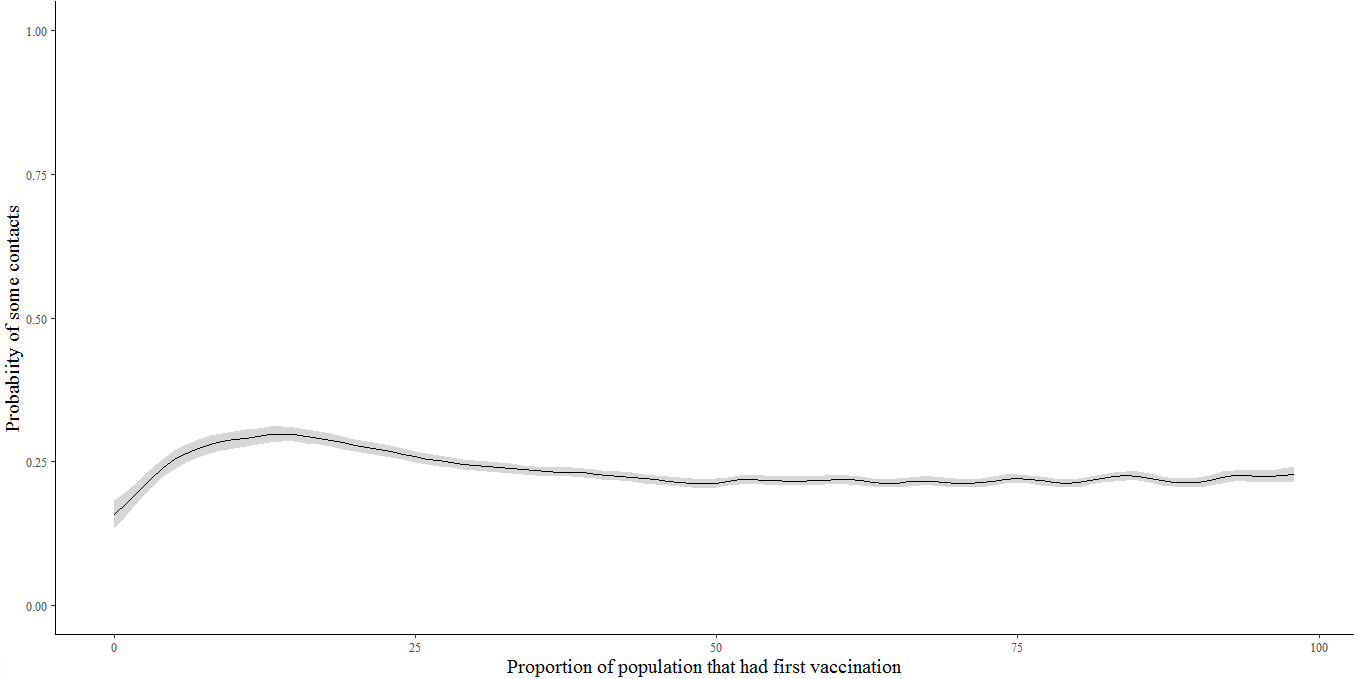


Supplementary figure 17: Probability of physical contacts18 to 64 with calendar time specified in weeks.


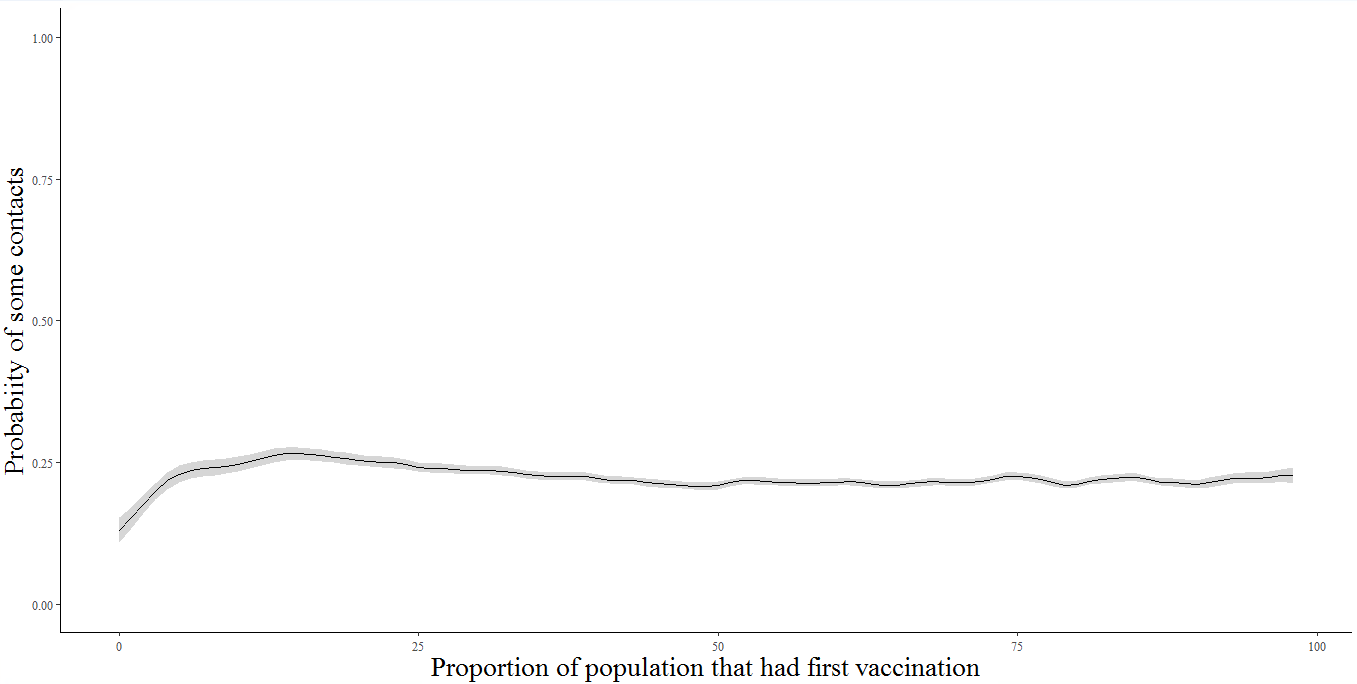


Supplementary figure 18: Probability of physical contacts18 to 64 with calendar time specified in fortnights.
